# Pushing the Frontiers: Artificial Intelligence (AI)‐Guided Programmable Concepts in Binary Self‐Assembly of Colloidal Nanoparticles

**DOI:** 10.1002/advs.202501000

**Published:** 2025-04-26

**Authors:** Cancan Li, Lindong Ma, Zhenjie Xue, Xiao Li, Shan Zhu, Tie Wang

**Affiliations:** ^1^ Tianjin Key Laboratory of Life and Health Detection Life and Health Intelligent Research Institute Tianjin University of Technology Tianjin 300384 China

**Keywords:** artificial intelligence, binary co‐assembly, colloidal nanoparticles, intelligent novel materials, self‐assembly, superstructural materials

## Abstract

Colloidal nanoparticle self‐assembly is a key area in nanomaterials science, renowned for its ability to design metamaterials with tailored functionalities through a bottom‐up approach. Over the past three decades, advancements in nanoparticle synthesis and assembly control methods have propelled the transition from single‐component to binary assemblies. While binary assembly has been recognized as a significant concept in materials design, its potential for intelligent and customized assembly has often been overlooked. It is argued that the future trend in the assembly of binary nanocrystalline superlattices (BNLSs) can be analogous to the ‘0s’ and ‘1s’ in computer programming, and customizing their assembly through precise control of these basic units could significantly expand their application scope. This review briefly recaps the developmental trajectory of nanoparticle assembly, tracing its evolution from simple single‐component assemblies to complex binary co‐assemblies and the unique property changes they induce. Of particular significance, this review explores the future prospects of binary co‐assembly, viewed through the lens of ‘AI‐guided programmable assembly’. Such an approach has the potential to shift the paradigm from passive assembly to active, intelligent design, leading to the creation of new materials with disruptive properties and functionalities and driving profound changes across multiple high‐tech fields.

## Graphical abstract

Colloidal nanoparticles self‐assembly advances towards intelligent, customized assembly through precise control of binary co‐assemblies. This review explores the evolution from monolithic to binary assemblies, highlighting how the AI‐guided programmable assembly approach has the potential to shift from passive assembly to active intelligent design. It paves the way for the development of disruptive new materials and profound changes in various high‐tech fields.

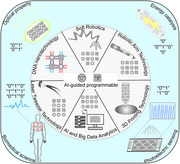

## 1 Introduction

Colloidal nanoparticles, defined as particles dispersed in a medium with at least one dimension at the nanometer scale, which can be either crystalline or amorphous in nature, are renowned for their exceptional optical, electrical, and magnetic properties. Typically, these particles consist of an inorganic core surrounded by a layer of material with specific functional groups serving as surface ligands. This coating can include, but is not limited to, long‐chain alkanes, polymers, or other organic and inorganic materials depending on the application requirements and design purposes.^[^ ^1^ ^]^ Advances in nanosynthesis technologies have enabled researchers to precisely control the size, chirality,^[^ ^2^ ^]^ and morphology^[^ ^3^ ^]^ of nanoparticles by fine‐tuning synthesis parameters, such as precursor selection, type of surface ligands, reaction temperature, and duration.^[^ ^1^, ^4^ ^]^ These control techniques not only facilitate the development of nanomaterials with unique properties but also lay the groundwork for designing novel composite materials and manufacturing functional devices. If a nanoparticle is likened to an ‘artificial atom’, then the monolithic assembly of nanoparticles can be analogously viewed as an “atom‐like assembly”, where each nanoparticle serves as an “atom‐like” unit.^[^ ^5^ ^]^ Arranging these units into a superstructure imitates the atomic arrangement in a crystal, resulting in well‐organized 1D, 2D, and 3D structures.^[^ ^6^ ^]^ Nano‐assembled structures not only inherit the fundamental properties of nanoparticles, such as the quantum confinement effect, size effect, and surface effect but also exhibit emergent phenomena arising from their unique configurations, such as quantum coupling and synergistic effects,^[^ ^7^ ^]^ opening a new pathway for synthesizing functional materials with desired collective properties by leveraging the weak interactions that exist at the nanoscale.^[^ ^8^ ^]^

As the understanding of nanoparticle self‐assembly mechanisms deepens and new materials are discovered, the field of colloidal nanoparticle self‐assembly continues to make significant breakthroughs.^[^ ^9^ ^]^ Research has shifted from the monocomponent assembly of single‐type nanoparticles to the binary assembly of different types of nanoparticles, leading to more complex functionalities and structures.^[^ ^6^, ^9^, ^10^ ^]^ Binary assembly typically involves combining nanoparticles with complementary properties, such as one providing stability and the other providing functionality.^[^ ^11^ ^]^ By precisely controlling the interactions between nanoparticles, researchers can design composite materials with specific optical, electrical, or bioactive properties.^[^ ^9^, ^10^, ^12^ ^]^ However, most current research efforts remain focused on synthesis methods,^[^ ^4^, ^10^, ^13^ ^]^ morphological characteristics,^[^ ^12^, ^13^, ^14^ ^]^ and basic properties^[^ ^4^, ^10^, ^13^, ^15^ ^]^ of BNLSs, and a variety of colloidal nanocrystalline self‐assembled materials have been designed and reported,^[^ ^1b,g^ ^5^, ^6^, ^8^, ^16^ ^]^ whereas the special properties and practical applications of these superstructures have been less explored. Moreover, the kinetic control of the traditional nanoparticle assembly process appears to be particularly insufficient. Due to the multitude of variables affecting the assembly process, including, but not limited to, temperature, concentration, solvent properties, types of surface modifiers and their coverage, etc., the complexity of these factors is intertwined, making the optimization of the assembly conditions extremely difficult. Against this background, researchers often need to spend a lot of time and effort on repeated experiments to find the best combination of parameters to achieve the desired assembly structure and function.

Recent advances in AI‐guided assembly strategies now offer powerful tools to address these limitations.^[^ ^10^, ^17^ ^]^ Introducing the concept of ‘AI programmable ideas’ is introduced into the binary assembly process of colloidal nanoparticles can provide a new perspective on assembly. Considering the binary co‐assembly of nanocrystals as a process analogous to computer programmable, where nanocrystal units are akin to the basic elements “0s” and “1s” in programmable languages, precise control over the positions and connections of these units, similar to writing a program, can enable the design of specific functional nanomaterials. Machine learning models trained on multimodal characterization data can accelerate the exploration of assembly parameter spaces through active learning algorithms, while physics‐informed neural networks enable the prediction of non‐equilibrium assembly pathways. This suggests the potential for a fundamental shift from traditional passive assembly to active intelligent design, while also introducing a new approach to material design: AI‐guided programmable assembly concepts into the design of nanomaterials.

**Figure 1:**
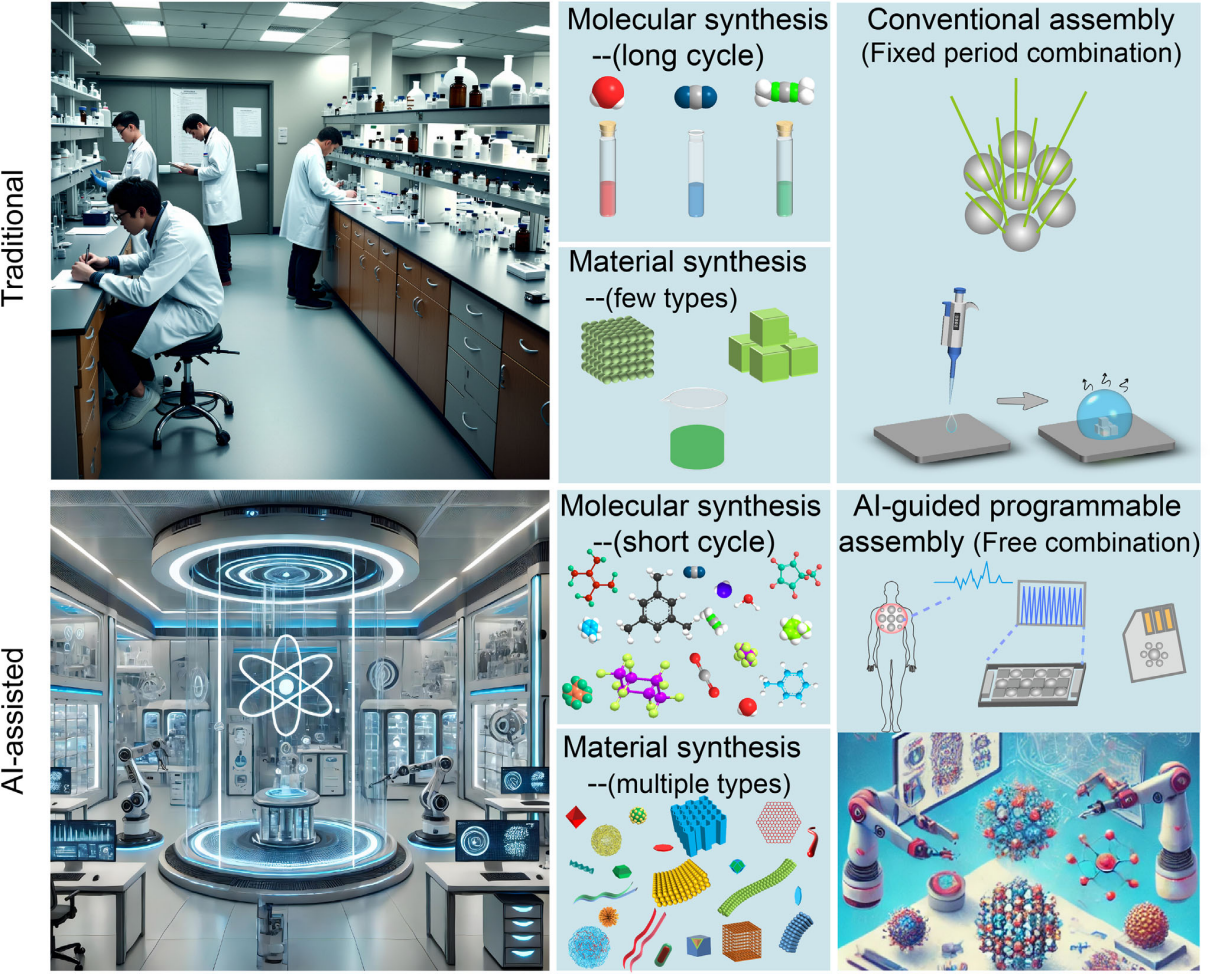
AI‐guided programmable idea self‐assembly of multiple colloidal nanocrystalline materials.

In this review, we briefly recap the developmental trajectory of nanoparticle assembly, tracing the property changes it has brought from initial single‐type nanoparticles to monocomponent assembly structures. Subsequently, we introduce the evolutionary process from monocomponent assembly to more advanced and complex binary nanoparticle assemblies combining different types of nanoparticles, focusing on exploring the unique properties of binary nanoparticle assembly and discussing the related application prospects it brings. Finally, we attempt to explore the future trends of colloidal nanoparticle binary co‐assembly technology from the unique perspective of AI‐guided programmable assembly (**Figure** **1**). By AI‐guided programmable assembly concepts, researchers can design nanomaterials with complex logical structures, thereby achieving a fundamental shift from passive assembly to active intelligent design. Through continuous exploration of new synthetic pathways, optimization of assembly strategies, and drawing inspiration from other disciplines such as information science and biology, it is possible to create more disruptive functional new materials. These new materials are poised to trigger profound changes in multiple fields, including energy conversion and storage, information processing and transmission, disease diagnosis and treatment, and beyond.

## 2 Colloidal Nanoparticle Self‐Assembly

Colloidal nanoparticle self‐assembly is a powerful technique for the bottom‐up fabrication of functional complex superstructures.^[^ ^18^ ^]^ The lower‐level assembly units of colloidal superstructures can have various forms, such as spherical,^[^ ^1^, ^9^, ^10^, ^19^ ^]^ polyhedral,^[^ ^1^, ^20^ ^]^ rod‐shaped,^[^ ^1^, ^12^, ^14^, ^21^ ^]^ and branched inorganic cores.^[^ ^1^, ^22^ ^]^ The physical and chemical properties of these units, as well as the modulation of their spatial arrangement through different assembly paths in the self‐assembled system, together determine the properties and characterization of the final superstructure.^[^ ^23^ ^]^ The collective properties of the superstructure are not only controlled by the characteristics of the lower‐level nanoparticles themselves but also influenced by the symmetry, orientation, phase, and size of the superstructure.^[^ ^8^, ^20^ ^]^ The regulation of the superstructure is achieved by controlling the interactions between colloidal nanoparticles, including but not limited to van der Waals forces, electrostatic interactions, and magnetic interactions.^[^ ^1^, ^7^, ^8^, ^24^ ^]^ By adjusting the strength and direction of these forces^[^ ^25^ ^]^, the arrangement of nanoparticles and the resulting structure can be effectively controlled. This method enables precise control over the spatial organization of nanoparticles into ordered superstructures, leading to the emergence of collective properties and specific functions that are not achievable with individual nanoparticles.

### 2.1 Methods of Colloidal Nanoparticles Self‐Assembly

**Figure 2:**
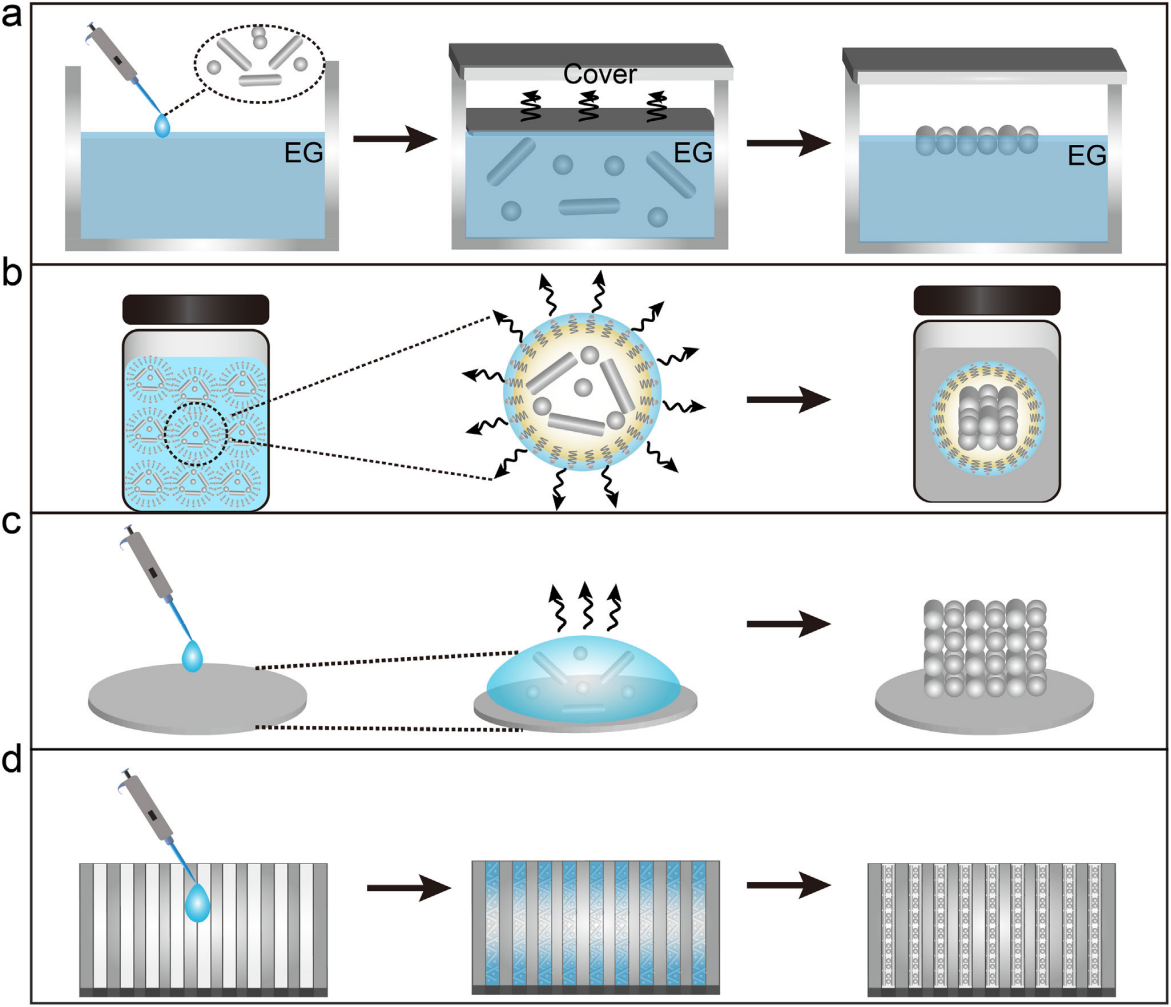
a) Schematic illustration of the self‐assembly mechanism at the gas‐liquid interface. b) Graphic representation of the microemulsion formation and assembly procedure. c) Visual depiction of the assembly process driven by solvent evaporation. d) Illustrative diagram of the induced self‐assembly mechanism.

Colloidal nanoparticle self‐assembly methods are the cornerstone for the preparation of metamaterials with different functions and structures. These methods, which mainly include gas‐liquid interfacial assembly^[^ ^9^, ^13^, ^26^ ^]^, microemulsion assembly, solvent evaporation assembly, and template‐induced assembly, utilize different physicochemical mechanisms to orchestrate the ordered arrangement of nanoparticles. Among them, the gas–liquid interfacial assembly method (**Figure** **2** **a**) uses the interfacial tension between the two phases to promote the self‐assembly of nanoparticles on the contact surface and the formation of an ordered structure. Microemulsion assembly involves both normal (oil‐in‐water) and reverse (water‐in‐oil) systems, where the interface between the two immiscible phases serves as a soft template. The size and stability of the microemulsion droplets (Figure 2b), controlled by surfactant concentration and type, determine the assembly process and final structure.^[^ ^27^ ^]^ Solvent evaporation assembly (Figure 2c) ^[^ ^28^ ^]^ directs the self‐assembly of nanoparticles into a highly ordered superlattice by controlling evaporation kinetics, environmental conditions (temperature and humidity), and substrate properties. Induced self‐assembly (Figure 2d), employs various guiding factors, such as physical templates (hard or soft) and external fields (electric, magnetic, or optical), to direct the spontaneous organization of nanoparticles into predetermined architectures.^[^ ^29^ ^]^

#### 2.1.1 Gas–Liquid Interfacial Assembly Method

The gas–liquid interfacial assembly method, as one of the earliest colloidal nanoparticle self‐assembly techniques, can be used to prepare superlattice structures, which is based on the principle of inducing nanoparticles to self‐assemble employing interfacial tension at the contact surfaces between an aqueous phase and a non‐aqueous phase (e.g., oil or air), which results in the formation of ordered structures.^[^ ^26a^ ^]^ This self‐assembly capability was made possible by advances in synthesis techniques that achieved near‐atomic‐level size uniformity of nanograins, allowing them to self‐assemble into ordered superlattice structures in a solution environment, similar to the formation of larger structures by atoms or molecules through chemical bonding. With the development of this method, researchers have proposed a series of improvements and strategies aimed at precisely regulating the morphology and size of the nano‐assembled materials,^[^ ^13c^ ^]^ such as controlling the arrangement of nanocrystalline grains at the interface by adjusting the solution concentration, the interfacial curvature, and the interaction force between the nanoparticles and the interface.^[^ ^6^, ^9^, ^15^, ^30^ ^]^

Of particular note is the work of Murray and colleagues,^[^ ^26a^ ^]^ who have made important contributions to this field. They showed how CdSe nanograins self‐assemble into a 3D quantum dot superlattice, which provides an important example of the self‐assembly of nanomaterials. Their study shows that by precisely controlling the size and spacing of the nanocrystals, an ordered arrangement of these nanostructures can be achieved and their unique optical and electronic properties further explored. Elena V. Shevchenko, Dmitry V. Talapin, and Nicholas A. Kotov, and colleagues have further applied this method to nanocrystal assembly and binary nanocrystal assembly.^[^ ^1^, ^10^, ^14^, ^24^, ^31^ ^]^ Their studies revealed that the superlattices formed by binary assembly of nanocrystals have many different structural forms, which provide new ideas and directions for the design and application of nanomaterials. Furthermore, focusing on the self‐assembly mechanism of nanocrystals at gas–liquid interfaces, Tobias Hanrath and his team have achieved large, homogeneous nanocrystalline layers through optimized treatments and have explored the optical and electrical properties of these structures, providing new routes and techniques for the fabrication of high‐performance electronic and optoelectronic devices.^[^ ^32^ ^]^ In addition, Vanmaekelbergh's team also demonstrated the self‐assembly of 2D honeycomb superlattices at the gas–liquid interface on the basis of specific crystalline surfaces between nanocrystals using the oriented attachment,^[^ ^33^ ^]^ a mechanism by which nanocrystals of PbSe, PbS, and, CdSe are oriented at interfaces to form honeycomb structures with long‐range periodic and atomically coherent honeycomb structure. These advances provide new routes for superlattice structure manipulation of a wide range of nanoparticles, including insulators, magnetic, and metallic materials, and open the way for the development of 2D semiconductor materials with novel electronic properties.

#### 2.1.2 Microemulsion Assembly Method

Microemulsion assembly method,^[^ ^5^, ^34^ ^]^ as a soft chemical technique widely used in the field of nanomaterials synthesis, forms water‐in‐oil (W/O) or oil‐in‐water (O/W) type microemulsions by dispersing nanoparticles in an oil phase and mixing them with the aqueous phase containing surfactants, using the oil droplets therein as the microreactor.^[^ ^27^ ^]^ Within the microemulsion system, the nanoparticles are aggregated, assembled, and finally immobilized inside the oil droplets through the hydrophobic van der Waals forces between the surfactant ligands adsorbed on the surface.^[^ ^34^ ^]^ As the low‐boiling oil solvent gradually evaporates in the restricted 3D space, the nanoparticles spontaneously aggregate and assemble into colloidal spheres with precisely controlled size, shape, and composition. This technique overcomes the limitations of traditional methods where only ‘soluble’ or dispersible aggregates of controlled size, shape, and composition can be prepared on substrates such as mica or silicon plates. Cao's team has reported multiple research focusing on the use of microemulsion self‐assembly techniques to achieve the assembly of nanoparticles with different morphologies, demonstrating the simplicity, broad applicability, ease of scalability, and ability to precisely control the size, shape, and composition of this approach.^[^ ^1^, ^5^, ^34^ ^]^

In addition, Alfons van Blaaderen and his group have explored in depth the microemulsion assembly of colloidal spheres by synthesizing a variety of nanoparticles and colloids into emulsions, which were solvent evaporated to achieve self‐assembly.^[^ ^35^ ^]^ The resulting colloidal superparticles exhibit a variety of structures depending on their size, and the formation of icosahedral clusters is entropy‐driven, which offers novel insights into cluster formation and self‐assembly mechanisms. Meanwhile, Nicolas Vogel and his team have further expanded the application of microemulsion assembly technology.^[^ ^23^, ^36^ ^]^ Their research not only focuses on the structural changes during colloidal self‐assembly, but also explores how these structures affect the functional properties of the final materials. For example, they have used microemulsions to prepare colloidal aggregates with specific optical properties, suggesting that this ‘structural coloring’ could be a tool for exploring structural and dynamic processes, and Vogel's research has also involved directing the crystallization process by controlling surface modifications of colloidal particles to enable the selection of crystal polymorphs, which could have implications for the development of novel functional materials. which is important for the development of novel functional materials. In addition, Andries Meijerink et al. further investigated the doping mechanism of hydrophobic quantum dots in silica spheres, and successfully prepared silica microspheres containing quantum dots by the reversed‐phase microemulsion method, which extends the scope of application of microemulsion assembly technology.^[^ ^37^ ^]^ This series of studies not only demonstrates the potential of microemulsion assembly in the preparation of complex structural materials but also provides a theoretical basis for understanding the self‐assembly process at the nanoscale.

Colloidal superparticles,^[^ ^5^, ^34^ ^]^ as an emerging building block, can exhibit both short‐range disordered amorphous features and long‐range ordered superlattice properties by the arrangement of nanoparticles inside them. These superparticles not only inherit the basic chemical and physical properties of the constituent nanoparticles but also exhibit entirely new collective properties due to the coupling of electrons, plasmons, and magnetism.^[^ ^1^, ^22^, ^38^ ^]^ In addition, due to their colloidal form, these superparticles can be easily assembled into mesoscopic and even macroscopic structures by solution treatment, showing great potential for applications in solar cells, light‐emitting diodes, and catalysts.

#### 2.1.3 Solvent Evaporation Method

The solvent evaporation method is widely used as an efficient preparation technique for the synthesis of colloidal nanoparticle superlattices.^[^ ^39^ ^]^ The core mechanism of this method is to induce the self‐assembly of nanoparticles into highly ordered structures through solvent evaporation.^[^ ^28^, ^40^ ^]^ In the specific operation, the pre‐synthesized and surfactant‐functionalized colloidal nanoparticles are first dispersed in a suitable solvent to form a solution, which ensures the stability of the nanoparticles in the solution and the ordering during the subsequent self‐assembly process.^[^ ^28^ ^]^ The choice of solvent plays a critical role in this process; solvents with specific physical and chemical properties, such as boiling point and polarity, significantly influence the self‐assembly behavior. Solvents with higher boiling points can slow down the evaporation rate, allowing for more controlled assembly processes, while the polarity of the solvent affects the interaction between nanoparticles and their environment, impacting how they align and aggregate. Moreover, the properties of the substrate surface, including its hydrophobicity/hydrophilicity and roughness, also have a profound impact on the self‐assembly of nanoparticles. A hydrophobic substrate tends to promote the formation of ordered superlattices by minimizing unwanted interactions with the solvent, whereas a hydrophilic substrate might lead to different assembly patterns or even disordered structures depending on the nature of the nanoparticles and the solvent. Additionally, the roughness of the substrate can affect the uniformity and quality of the assembled structure. Smooth substrates facilitate the creation of defect‐free, highly ordered assemblies, while rough surfaces may introduce defects or variations in the arrangement of nanoparticles.

After preparing the nanoparticle solution, it is deposited onto a selected substrate, and gradual solvent evaporation is induced by modulating environmental parameters like temperature and humidity. With the slow evaporation of the solvent, the distance between the nanoparticles gradually shrinks, which ultimately leads to the enhancement of inter‐particle attraction and self‐assembly into a superlattice structure.^[^ ^41^ ^]^ Precise control of the solvent evaporation rate is crucial in this process, as too fast an evaporation rate may lead to random deposition of nanocrystalline particles instead of the expected ordered arrangement. Zhao et al. found that when a suspension of gold nanoparticles (GNPs) containing excess oleic acid (OA) ligand is dried on a hydrophobic substrate,^[^ ^42^ ^]^ the nanoparticles undergo a series of transitions from disordered stacking to ordered superlattices. The key to this transition is the aggregation behavior of the nanoparticles at the evaporation front due to the rapid withdrawal of the solvent during evaporation. This series of observations provides important insights into understanding and controlling the ordered assembly of nanoparticles.

#### 2.1.4 Induced Assembly Method

**Figure 3:**
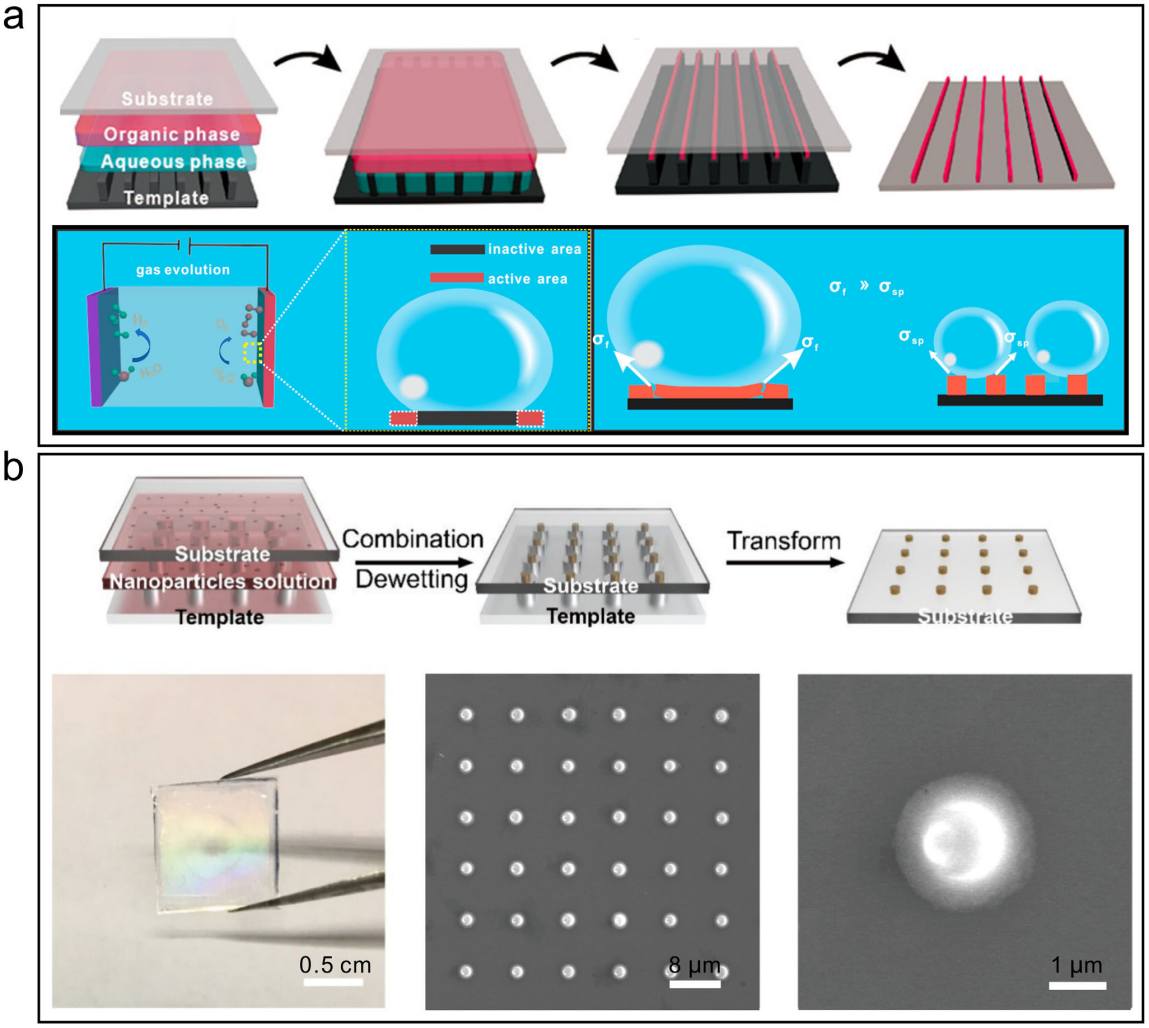
a) Schematic illustration of the method for fabricating SP superlattices using the template‐induced self‐assembly and the schematic illustration of the growth of gas bubbles on a flat film electrode, which caused a large number of inactive sites to form. Reproduced with permission.^[^ ^43^ ^]^ Copyright 2019, ACS. b) Fabrication of patterned AuNP superlattice film by template‐induced self‐assembly. Reproduced with permission.^[^ ^44^ ^]^ Copyright 2024, ACS.

Induced self‐assembly is a process that uses external factors to guide the spontaneous assembly of nanocrystalline materials into ordered structures. By precisely regulating external conditions or introducing specific templates, the self‐assembly process can be highly directed and controlled to prepare nano‐assemblies with specific geometries, structural features, and functional properties. The template‐induced nanoparticle self‐assembly method has many advantages, such as the ability to precisely control the morphology and structure of the nanoparticles, ease of mass production, and reproducibility.^[^ ^29^ ^]^ Song et al. report a general strategy (**Figure** **3**a) for assembling platinum (Pt) NPs into striped superlattices via template‐assisted printing to improve the efficiency of the hydrogen evolution reaction (HER).^[^ ^43^ ^]^ Compared to flat Pt NPs films prepared by the drop‐coating method, the striped superlattices not only improved the mass transfer efficiency but also reduced the bubble stretching force, demonstrated a versatile strategy to improve the efficiency and durability of existing Pt catalysts, and demonstrated higher current densities than those of commercial Pt/C, Pt NPs films, and other Pt‐based or non‐Pt‐based HER catalysts reported in the literature. The versatility of the template‐assisted printing technique allows flexibility in the composition, size, and shape of the nanoparticles or molecules, thus extending the scope of this accelerated technique for oxygen evolution reaction (OER) and electrochemical reduction of carbon dioxide to carbon monoxide applications. Meanwhile, we prepared AuNP superlattice films using a template printing method (Figure 3b) for the construction of wearable sweat sensors.^[^ ^44^ ^]^ By adjusting the size of the AuNP superlattice domains below the fracture critical size, the mechanical stability was improved, and a long‐term, reliable high‐performance signal output was achieved. These two works not only demonstrate the potential of template assembly techniques in improving catalyst performance but also reveal that improvements in the mechanical stability of materials can be achieved by controlling the way nanomaterials are assembled.

### 2.2 Characterization of Colloidal Nanoparticle Superstructures

The development of characterization techniques has laid a solid foundation for the study of nanoparticle superstructures. From traditional scanning transmission electron microscopy (STEM) to today's cryogenic electron tomography (cryo‐ET), liquid‐phase transmission electron microscopy (LPEM), as well as the integration of advanced image processing and reconstruction algorithms, these technological advancements have made it possible to characterize the 3D structures of nanoparticles in conditions close to their natural environments in detail. Among them, LPEM allows for real‐time observation of nanoparticles in a liquid medium, which is crucial for studying processes like self‐assembly and nanoparticle‐liquid interactions that occur in liquid environments.^[^ ^45^ ^]^ One particularly important method is electron tomography, which is used to form a 3D model of a sample by taking 2D projected images from multiple angles and combining these images using computational methods.^[^ ^46^ ^]^ This technique has the advantage of high resolution and 3D reconstruction, allowing structural features to be resolved at the nano‐ or even sub‐nanometer scale, and is particularly suitable for the study of nanomaterials with complex internal structures or those that are difficult to analyze by other methods. Traditional STEM techniques are limited by the vacuum environment and struggle to reflect the true state of nanoparticles in their natural liquid settings. Cryogenic electron tomography overcomes issues related to solvent evaporation or changes in 3D configuration due to contact with support grids by rapidly freezing samples to preserve their original state. However, subtle structural changes may still occur in experimental settings.

To address these issues, Sara Bals and her team have made particularly notable contributions by developing liquid‐phase fast electron tomography (LP fast electron tomography),^[^ ^47^ ^]^ solving the problem of structural distortion caused by solvent evaporation or capillary forces in a vacuum environment, thus allowing for 3D characterization studies of colloidal assemblies in water or various solvents. Initially, using a K‐kit liquid cell system and robust principal component analysis (RPCA) methods, researchers were able to efficiently acquire and process 3D structural data of samples in liquids.^[^ ^47^ ^]^ They optimized image registration through the iterative closest point (ICP) algorithm and addressed the missing wedge issue caused by a limited angular range with a novel 3D reconstruction algorithm. Research shows that this technology enables detailed structural analysis of nanoparticles while maintaining their native state, providing a powerful new tool for exploring biomolecules, soft matter, and nanomaterials.

Initial attempts used amorphous silicon nitride microfluidic chambers as liquid cells, but reconstruction accuracy was compromised due to inadequate angular sampling. The application of graphene liquid cells (GLCs) significantly improved the signal‐to‐noise ratio (SNR) and allowed for the study of growth,^[^ ^47^ ^]^ self‐assembly, and dynamic processes of nanomaterials. Through the use of high‐quality GLCs, researchers observed that CTAB ligands do not form conventional bilayer structures but may exist in micellar form, exhibiting dynamic characteristics in a liquid environment, which differs from observations under dry conditions. This work not only provides new insights into the local structure and distribution of CTAB on the surface of individual gold NRs but also proposes a new 3D reconstruction algorithm to solve the missing wedge problem caused by a limited angular range, highlighting the shortcomings of traditional reconstruction algorithms.

In the field of structural characterization of colloidal nanoparticles, computer simulation plays a crucial and multidimensional role. First, computer simulation can assist in predicting the formation of complex structures, and the algorithms developed by Glotzer and Engel et al. have the ability to predict the structure of self‐assembled polyhedral nanoparticles, allowing researchers to know in advance what structures are likely to emerge, and thus allow them to carry out experimental studies in a more targeted manner.^[^ ^48^ ^]^ Focusing on the areas of dense stacking of crystals and disordered materials, Torquato and team have provided a theoretical basis for understanding the stacking mode of polyhedra and the formation of crystal structures, which is an important guide for material design and structural studies in related fields.^[^ ^49^ ^]^ Second, computer simulation and experiment verify each other, and together they construct a closed‐loop optimization system of ‘simulation guides experiment‐experiment verifies simulation’. Taking the research of Dijkstra and his co‐workers as an example, they used theoretical calculations and Monte Carlo simulation methods to thoroughly investigate the process of self‐assembly of colloidal hexagonal biconical and double‐truncated conical ZnS nanocrystals into a 2D superlattice, and found that small truncations change the symmetry of the superlattice. In addition, they showed experimentally and through simulations that entropy and spherical confinement are sufficient for the self‐assembly of hard spheres into icosahedral clusters.^[^ ^35^, ^50^ ^]^ Furthermore, Vanmaekelbergh and his team successfully revealed the formation of 2D PbSe superstructures with the help of in‐situ grazing incidence X‐ray scattering, non‐in‐situ electron microscopy, and Monte Carlo simulations.^[^ ^51^ ^]^

In addition, computer simulations have helped to investigate the self‐assembly of different nanoparticles, and Manna's team has found that octopod nanocrystals can self‐assemble into linear chains and further form 3D superstructures through a combination of experiments and simulations.^[^ ^52^ ^]^ Yang et al. demonstrated experimentally and through computer simulations that nanoscale Ag polyhedra can self‐assemble into the putative most densely packed structures, and even into superstructures with complex helical patterns.^[^ ^53^ ^]^ Chen et al. on the other hand, combined liquid‐phase transmission electron microscopy with computational modelling to reveal the control of the thermodynamic equilibrium by van der Waals forces and electrostatic interactions during the self‐assembly of tetrahedral gold nanoparticles, as well as the mechanism by which the fine‐tuning of the chirality of the variable corner‐to‐corner connections is achieved.^[^ ^54^ ^]^ In summary, the computational tools in these studies greatly accelerate the structure resolution process, successfully establish a closed‐loop optimization system of ‘simulation guides experiment‐experiment verifies simulation’, and strongly promote the development of the field of colloidal nanoparticle structure characterization.

In summary, advancements in characterization technologies, including but not limited to cryo‐ET, LPEM, and advanced image processing and reconstruction algorithms, have provided us with a powerful toolbox for thoroughly investigating the 3D structures and dynamic behaviors of nanoparticles under conditions close to their natural environment. This not only deepens our understanding of the self‐assembly mechanisms of nanoparticles but also provides theoretical support and technical assurance for the design of new types of nanomaterials. In the future, continuous optimization and improvement of these technologies are expected to achieve a more precise and comprehensive 3D analysis of nanoparticle superstructures.

### 2.3 Colloidal Nanoparticle Superstructure

The assembled superstructures of colloidal nanoparticles can be classified into two categories based on their degree of spatial and orientational order: crystalline superstructures and amorphous superstructures. Each type of superstructure offers distinct advantages for specific applications. Crystalline superstructures usually refer to nanoparticles arranged in a long‐range ordered manner to form a superlattice similar to a crystal structure,^[^ ^24^, ^55^ ^]^ which exhibits unique optical, magnetic, and electrical properties. In contrast, amorphous superstructures, in which nanoparticles are arranged in a disordered or short‐range ordered manner, lack long‐range periodicity but can achieve specific functional properties by tuning their local ordering, and exhibit significant advantages in applications that require mechanical properties or a broad spectrum of homogeneous optical response from the material.^[^ ^56^ ^]^

#### 2.3.1 Crystalline Superstructures

Crystalline superstructures are material systems with long‐range ordered structures formed by the self‐assembly of colloidal nanoparticles, in which the nanoparticles follow a certain regular arrangement and exhibit periodic crystal‐like structures, also known as superlattice materials.^[^ ^24^, ^57^ ^]^ At the heart of building such crystalline superstructures lies the precise modulation of the interaction forces between nanoparticles to induce them to self‐organize at specific locations, leading to the formation of highly ordered superlattices. This process requires fine control of multiple factors, such as the surface properties of the nanoparticles, the environmental conditions of the solution, and external stimuli, to ensure that the nanoparticles are arranged according to predefined rules, thus generating advanced ordered materials with periodic structures.^[^ ^58^ ^]^ The study of superlattice materials not only deepens the knowledge in the field of fundamental optical science but also promotes the development of cutting‐edge applications, including optoelectronic devices and biosensing.

##### Optical Properties

**Figure 4:**
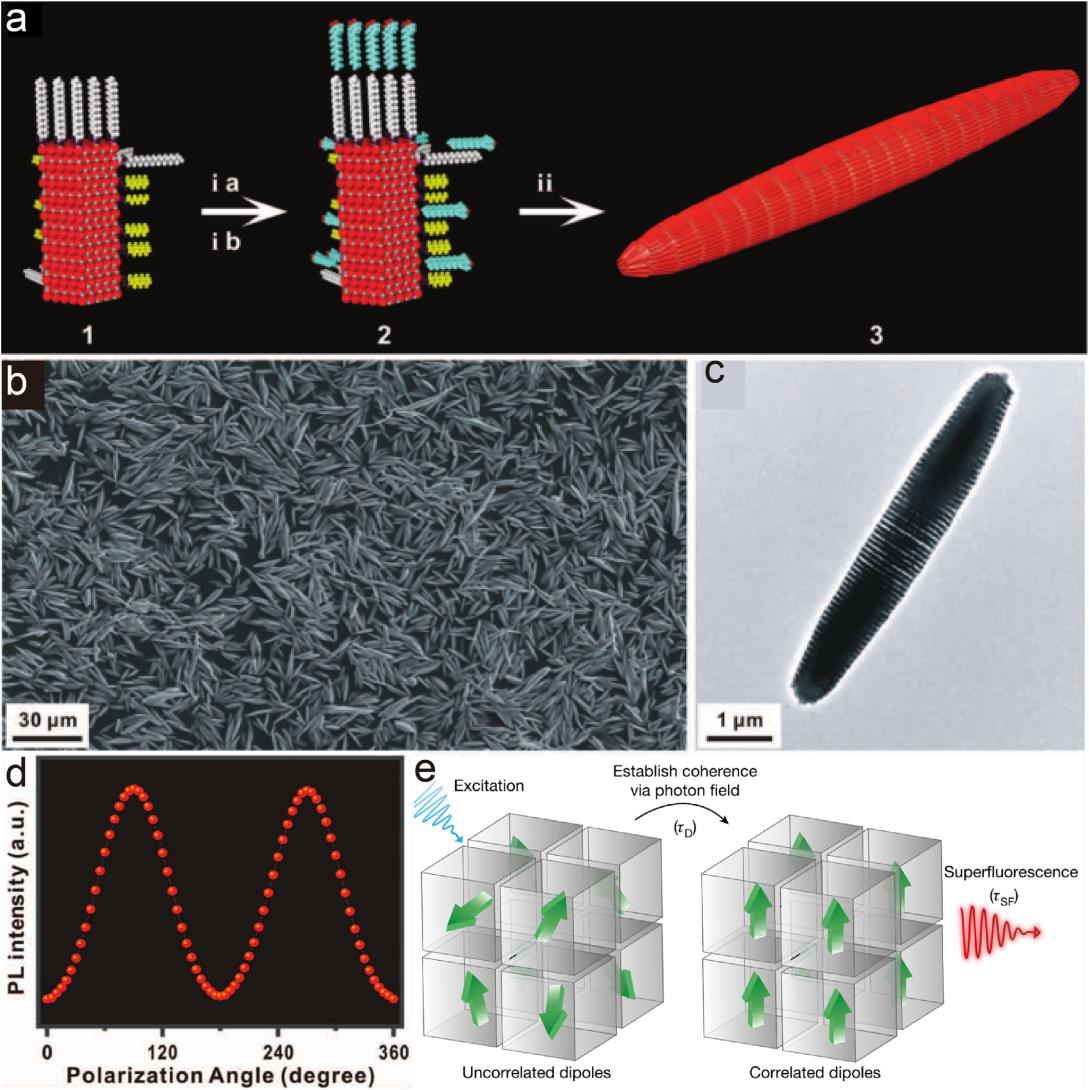
a) Scheme of needle‐like superparticle synthesis. b–c) Scanning electron microscope (SEM) image and TEM image of needle‐like superparticle. d) PL intensity versus polarization angle as the polarization was manually rotated while measuring a typical superparticle‐embedded PDMS thin film under the excitation wavelength of 380 nm. Reproduced with permission.^[^ ^1c^ ^]^ Copyright 2012, AAAS. e) Schematic of the build‐up process of superfluorescence. Reproduced with permission.^[^ ^7b^ ^]^ Copyright 2018, Springer Nature.

Superlattice materials show great potential for optical properties due to their unique energy band engineering and quantum effects.^[^ ^1^, ^35^, ^59^ ^]^ Through in‐depth study of the energy band structure and quantum effects of superlattice materials, scientists can design new materials with excellent optical properties suitable for light‐emitting devices such as light‐emitting diodes and lasers.^[^ ^1^, ^26^ ^60^ ^]^ In 2009, Cao's research team reported a self‐assembly study of anisotropic cadmium selenide/cadmium sulfide (CdSe/CdS) NRs.^[^ ^34^ ^]^In the presence of different ligands, the CdSe/CdS nanorods formed colloidal superparticles in cylindrical disks or stacked disk arrays, which exhibited linearly polarized photoluminescence properties along the axial direction. The superlattice structure can finely modulate the energy band structure of electrons and holes, thus affecting the carrier injection, transport, and compounding processes, and thus optimizing the luminous efficiency and other performance parameters of light‐emitting diodes. In 2012, additional research expanded our understanding of the assembly of colloidal superparticles. These studies demonstrated that it was possible to create colloidal superparticles with multiple well‐defined superlattice domains by leveraging the geometric morphology and structural orientation inherent to nanorods. By introducing kinetic anisotropic interactions based on functional requirements, scientists were able to successfully prepare single‐domain needle‐like superparticles (**Figure** **4**a–c).^[^ ^1^ ^]^ Notably, these superparticles exhibited optoelectronic properties that were linearly polarized along their axial direction (Figure 4d). This finding suggested potential applications in areas such as polarized light‐emitting diodes (PLEDs) and electro‐optical modulators, highlighting the promise of these materials in advanced optoelectronic devices.

It has been found that lead‐halide chalcogenide quantum dots exhibit hyperfluorescence when they self‐organize to form a 3D‐ordered superlattice (Figure 4e).^[^ ^7^ ^]^ The study shows that under high excitation density conditions, these chalcogenide nanoparticles exhibit key features of superfluorescence such as dynamic red‐shifted emission, radiative decay with more than 20‐fold acceleration, more than the fourfold extension of the first‐order coherence time, photon clustering effect, and Burnham‐Chiao oscillatory behavior. This discovery heralds potential applications in the field of high‐performance optoelectronic devices and multiphoton quantum light sources and is expected to promote the development of long‐distance quantum information transmission and ultra‐narrow tunable lasers. In addition, it was found that the long‐chain sulfobetaine‐capped CsPbBr₃ nanoparticles also exhibit monodispersity and hyperfluorescent assembly properties.^[^ ^61^ ^]^ The study also developed theoretical models to explain the size dependence of the first and second exciton jumps in the anisotropic shapes and absorption spectra of the nanoparticles, while elucidating the exciton lifetimes at room temperature. This advancement helps to reveal the fundamental photophysical properties of such nanoparticles and promotes their applications in optoelectronic devices and quantum optics. These remarkable properties not only position superlattice materials as an ideal and indispensable choice for the cutting‐edge domains of modern optical technology and quantum information science but also provide a new direction for the development of new materials, which further promotes the progress of related technologies.

##### Photoelectrical Properties

Due to its special periodic arrangement and interfacial properties, the superlattice structure is indeed capable of absorbing incident photons efficiently and separating carriers effectively, thus exhibiting excellent optoelectronic properties.^[^ ^13^ ^]^ For example, the use of PbS as superlattice materials in infrared detectors has demonstrated excellent performance;^[^ ^62^ ^]^ and silicon‐based superlattice structures have also been extensively studied in the visible range.^[^ ^63^ ^]^ By adjusting the parameters such as layer thickness, material combination, and doping level, the response of the sensor to specific wavelengths of light can be optimized, which gives it a broad application prospect in the fields of environmental monitoring, biomedical detection, and communication technology.

**Figure 5:**
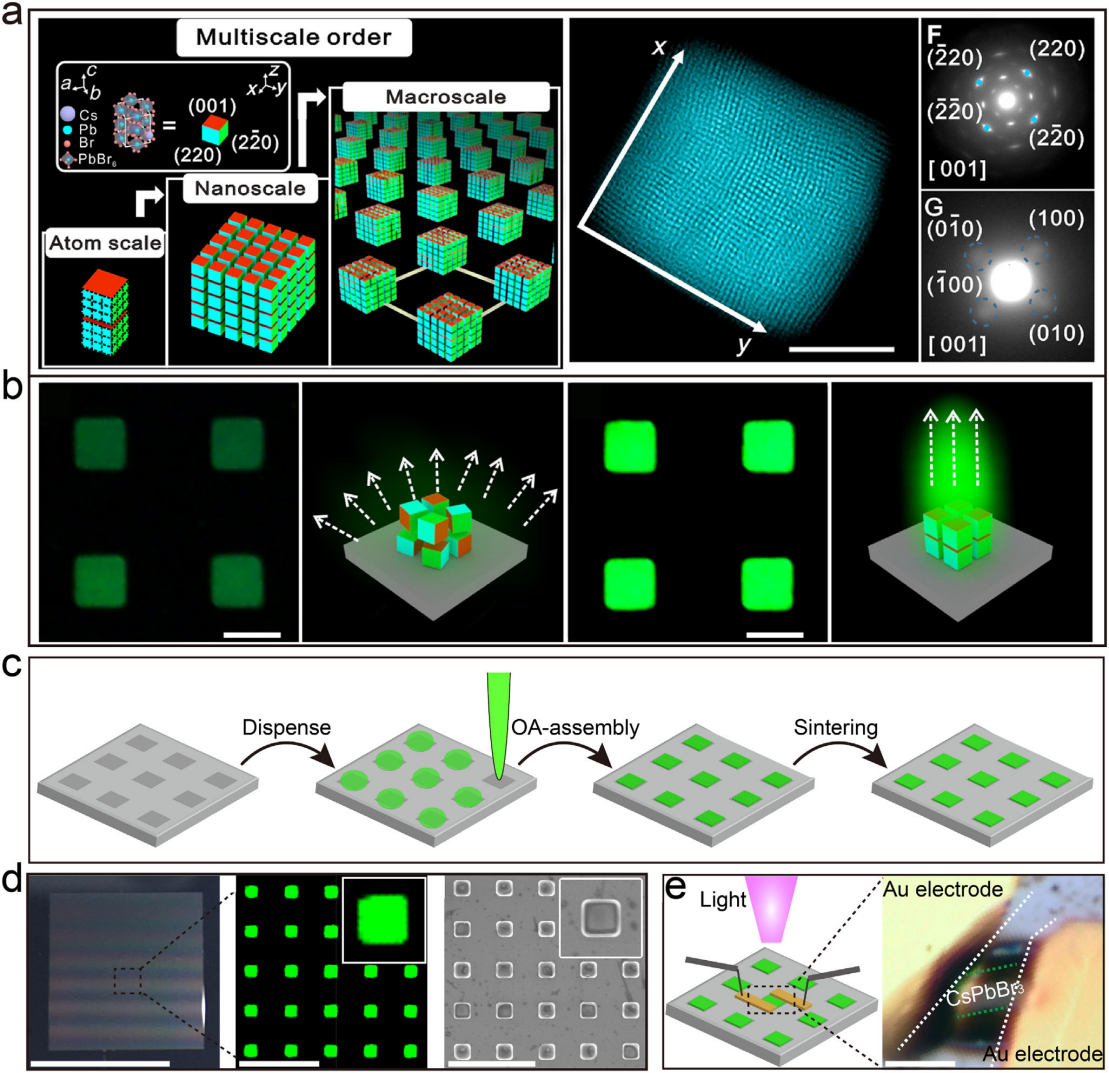
a) Schematic diagram of a single superlattice projected along the z direction and high‐angle toroidal dark‐field scanning transmission electron microscopy image. Scale, 200nm. b) Preparation process of superlattice array. Reproduced with permission.^[^ ^20^ ^]^ Copyright 2022, AAAS. c) Schematic of the preparation of the CsPbBr_3_ single‐crystal microstructure array via NSALS. d) Detailed characterization of the CsPbBr_3_ single‐crystal microstructure array. e) Schematics of the CsPbBr_3_ single‐crystal microstructure array photodetector and the optical microscopy image of the photodetector prepared using a gold patch electrode; scale bar: 5 µm. Reproduced with permission.^[^ ^64^ ^]^ Copyright 2024, ACS.

Meanwhile, perovskite nanoparticles have attracted much attention in the field of optoelectronic devices due to their unique optoelectronic properties, especially when they form a superlattice structure, where their optical and electronic properties are significantly enhanced. Li et al.^[^ ^20^ ^]^ demonstrated that by precisely controlling the self‐assembly process, a large‐scale ordered arrangement of CsPbBr_3_ perovskite nanocubes can be achieved to form superlattice arrays with excellent optical properties (**Figure** **5**a). These nanocubes self‐assembled into a superlattice exhibited a diffraction pattern similar to that of single crystals on the microscopic scale, and their fluorescence intensity was 5.2 times higher than that of randomly arranged nanocube arrays. In addition, the team demonstrated how to prepare a micropixel luminescent layer containing arrays of nanocubic superlattices acting as primary color photon emitters on a meter‐scale panel by printing technology (Figure 5b), foreshadowing its potential application in the development of high‐performance opto‐electronic devices and highly efficient directional quantum light sources. Based on these studies, we further improved the assembly method and developed a low‐temperature sintering technique assisted by nanoparticle self‐assembly (Figure 5c),^[^ ^64^ ^]^ which led to the highly ordered arrangement of perovskite nanostructures and the successful preparation of single‐crystal microstructures (Figure 5d). With the help of atomically oriented superlattice templates formed by nanoparticle self‐assembly, we were able to grow single‐crystal CsPbBr_3_ microstructures on different substrates, which exhibit consistent vertical orientation, extended carrier lifetime, and rapid, sensitive, and reproducible optical response, making them ideal for use in high‐performance photodetectors and optical sensing microarray chips (Figure 5e). This technology not only provides a new path for large‐scale production of high‐quality perovskite single‐crystal materials but also enhances the flexibility and broad applicability of chalcogenide materials in optoelectronic device integration and practical applications.

##### Raman Enhancement Effect

**Figure 6:**
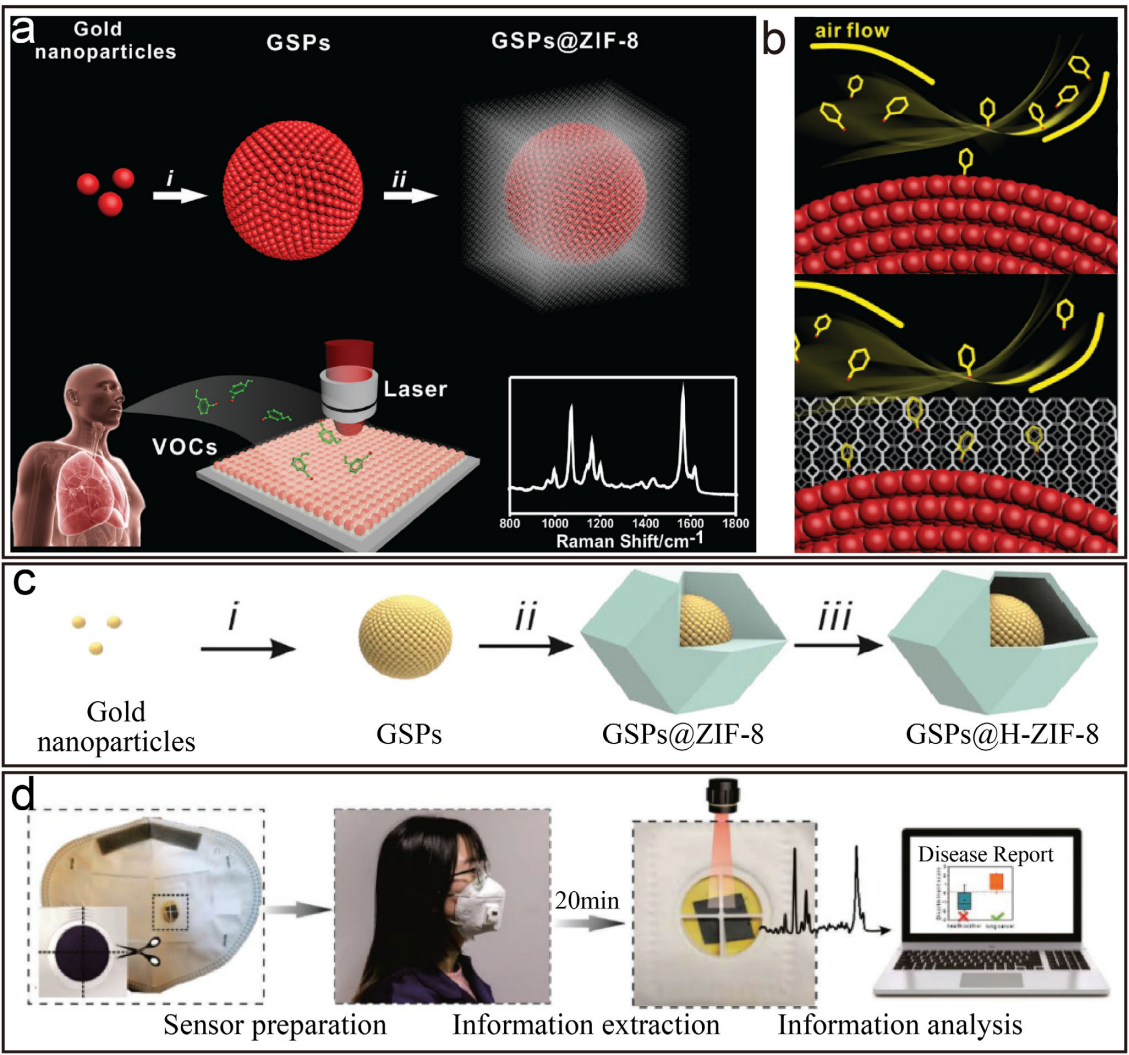
a) Schematic diagram of the preparation of GSPs@ZIF‐8 and schematic diagram of the detection of volatile organic compound (VOC) via surface‐enhanced Raman scattering (SERS) spectroscopy. b) Schematic illustration of GSPs and GSPs@ZIF‐8 with gas collisions. Reproduced with permission.^[^ ^1^ ^]^ Copyright 2018, Wiley‐VCH. c) Schematic diagram of the synthetic route of GSPs@H‐ZIF‐8. d) Diagram of a SERS sensor for VOC detection and digital photos of paper‐based SERS substrate and mask sensing device for the breath test. Reproduced with permission.^[^ ^66^ ^]^ Copyright 2022, Wiley‐VCH.

In addition to possessing excellent optoelectronic properties, superlattice materials have demonstrated great potential for the Raman enhancement effect. The successful application of superlattice structures in biosensing relies on the precise modulation of the size, shape, and surface chemistry of their constituent nanoparticles. When these parameters are optimized, superlattice materials can achieve amplified signals, thereby enhancing the sensitivity of biosensors.^[^ ^65^ ^]^ For instance, research conducted by Qiao et al. demonstrated the highly sensitive and selective detection of lung cancer biomarkers in exhaled breath using the enhanced Raman effect of assembled GNPs, known as gold superparticles (GSPs) (**Figure** **6**a).^[^ ^1^ ^]^ Due to the ordered and precise arrangement of AuNPs, coating the surface of gold superparticles with ZIF‐8 exhibited strong Raman enhancement, which enhanced the Raman signal of the analyte aldehyde molecules. The experimental results showed that the structure could track gaseous aldehyde molecules (Figure 6b), indicating significant application prospects. To further enhance the performance of the structure, Li et al. designed an egg yolk‐shell‐like hollow GSPs@ZIF‐8 (Figure 6c).^[^ ^66^ ^]^ Compared to the previous structure, this design not only maintained the effect of Raman enhancement but also enriched the gas through the hollow layer ZIF‐8, producing a stronger response (Figure 6d). Moreover, the structure selectively excluded interfering molecules, leading to a dramatic reduction in the detection limit and demonstrating immense potential in human exhaled breath detection.

Emerging advances in nanomaterial assembly have positioned monolithic architectures as focal points in materials science, driven by their exceptional physicochemical characteristics and versatile application potential. However, despite demonstrating remarkable capabilities, single‐component systems face intrinsic limitations in functional diversity and adaptability to dynamic operational demands, restricting their utility in multifaceted real‐world scenarios. This critical gap has catalyzed a strategic shift toward binary assembly frameworks, where two distinct nanomaterials are synergistically integrated. By harnessing interfacial synergy, such systems transcend the inherent constraints of monolithic designs, merging complementary attributes of individual components while generating emergent properties unattainable in isolated materials. These synergistic interactions not only address scalability challenges but also enable programmable customization of material behaviors for targeted applications. The following section will critically analyze contemporary breakthroughs in binary assembly methodologies, with emphasis on structure‐property correlations and performance optimization strategies.

## 3 Binary Co‐assembly of Colloidal Nanoparticles

With the continuous progress of nanosynthesis technology, the binary co‐assembly of different types of colloidal nanoparticles according to specific design principles to achieve more complex functions and structures has attracted extensive attention.^[^ ^9^, ^10^, ^14^, ^67^ ^]^ Compared with single types of nanoparticles, the binary assembly of colloidal nanoparticles offers several potential advantages. First, the contact interfaces between different nanoparticles exhibit unique chemical and physical properties, which are important for a deeper understanding of interfacial effects and interfacial reaction processes. Second, the performance of optoelectronic devices can be optimized through the integration of nanoparticles with diverse optical and electronic properties. Additionally, the self‐assembly of binary nanoparticles can regulate both the interfacial reactions and the photoelectron transfer behaviors involved in photocatalytic reactions and photosensing processes, thereby enhancing photocatalytic activity and photosensing performance. Finally, the co‐assembly of two nanoparticles with complementary electronic properties provides the possibility of designing and fabricating new types of electronic devices, such as nanocrystalline transistors, nano‐circuits, and nano‐sensors. This approach opens up new paths and methods for the development of the field of nanoelectronics, demonstrating great potential in practical applications.

### 3.1 Binary Co‐Assembly of Spherical Colloidal Nanoparticles

In more than two decades of research on the binary self‐assembly of colloidal nanoparticles, most efforts have focused on the two‐sphere system.^[^ ^9^, ^10^, ^13^, ^31^, ^68^ ^]^ In this context, Murray's group has made significant contributions, advancing the field substantially. In 2003, they reported the first binary superlattice composed of two groups of nanoparticles with size‐controllable properties.^[^ ^9^ ^]^ Each group could interact with or couple to neighboring particles with diameters of less than 2 nm. The increasing availability of monodisperse nanocrystalline systems has facilitated the development of a wide range of materials, including various BNLSs. In 2006, further studies using semiconducting, metallic, and magnetic nanoparticles as building blocks successfully synthesized more than fifteen different BNLSs ^[^ ^9^ ^]^ These studies revealed that nanoparticle surface charges determine the stoichiometric ratios of these superlattices and that entropic, van der Waals, spatial repulsive, and dipolar forces are crucial factors in their formation. By 2010, researchers had achieved the rapid growth of large‐scale, well‐ordered BNLS films using a method involving the co‐crystallization of multi‐component nanoparticles at the liquid‐gas interface.^[^ ^13^ ^]^ The scalability of this technique greatly facilitates the fabrication of nanocrystalline devices and holds promise for accelerating further exploration of these novel materials.

Subsequently, Talapin's research team focused on the study of the assembly mechanism and explored the role of hydrocarbon ligands in the self‐assembly of nanocrystalline superlattices through a series of experiments.^[^ ^9e^ ^]^ They demonstrate the deformability of the ligand overlayer, a property that leads to efficient tuning of the nanoparticle size in response to changes in the coordination environment. The role of the ligand corona in the thermodynamics and kinetics of the formation of BNLS assemblies was further elucidated by a set of systematic experiments.^[^ ^31c^ ^]^ Using gold (Au) and lead sulfide (PbS) nanoparticles containing hydrocarbon ligands as a model system, the researchers systematically tuned the core radius (R) and ligand chain length (L) of the particles and assembled them into binary superlattices. The resulting binary structure database allows for a detailed analysis of the role of the effective nanoparticle size ratio as well as the softness, expressed in terms of L/R, in guiding the binary structure assembly. This superlattice library allows not only the study of the frequencies of different phases but also the systematic measurement of the geometrical parameters of the superlattice. Based on this analysis, the researchers evaluated new theoretical models dealing with the co‐crystallization of deformable spheres and proposed new hypotheses on the factors affecting the nucleation and growth of binary superlattices.

### 3.2 Binary Co‐assembly of Anisotropic Colloidal Nanoparticles

In contrast to the study of spherical nanomaterials, binary self‐assembly explorations involving anisotropic nanomaterials, although emerging at a later stage, have witnessed remarkable progress in recent decades.^[^ ^67^, ^69^ ^]^ These studies have not only demonstrated the potential for more precisely orientated assembly and interaction between two colloidal nanomaterials with different shapes and compositions but also revealed new mechanisms to control the formation of nanoscale structures. By finely tuning the interactions between nanoparticles and their ratios, such as by using surface modification and introducing functional molecules, researchers have opened up new avenues for nanomaterial assembly.^[^ ^9^, ^10^, ^19^, ^70^ ^]^

Anisotropic nanomaterials exhibit more complex interaction patterns due to their unique geometries and compositional differences. It is found that the binary combination of spherical metal nanoparticles with CdSe/CdS nanorods is kinetically limited and that suitable additives and spherical nanoparticles with high dielectric constants and large Hamaker's constants have a significant effect on the formation process.^[^ ^14b^ ^]^ Subsequently, Murray's group combined experimental and computational approaches to investigate how nanorods can overcome the natural entropic tendency towards macroscopic phase separation and aggregate into three distinct phases on the centimeter scale.^[^ ^14c^ ^]^ Monte Carlo simulations indicated that while the alloy is entropically stable at high stacking fractions, it tends to phase‐segregate at experimental densities. The simulations also revealed that short‐range attractive forces, induced by ligand stabilizers and/or depletion effects, could stabilize the alloy structure. In 2015, the group further demonstrated the successful construction of binary and ternary superlattices by combining colloidal 2D LaF₃ nanodiscs with 1D CdSe/CdS nanorods via a liquid‐phase interfacial assembly technique.^[^ ^12^ ^]^ By regulating the liquid‐phase subenvironment used in the self‐assembly process, the researchers achieved effective tuning of the macroscopic orientation of the superlattices and ultimately prepared lamellar binary liquid crystal superlattice structures. This series of studies not only enriches our understanding of the self‐organization behavior of nanomaterials but also provides the theoretical basis and technical means for the design of novel functional materials.

### 3.3 Properties and Applications of BNLSs

#### 3.3.1 Optical Collective Phenomenon

**Figure 7:**
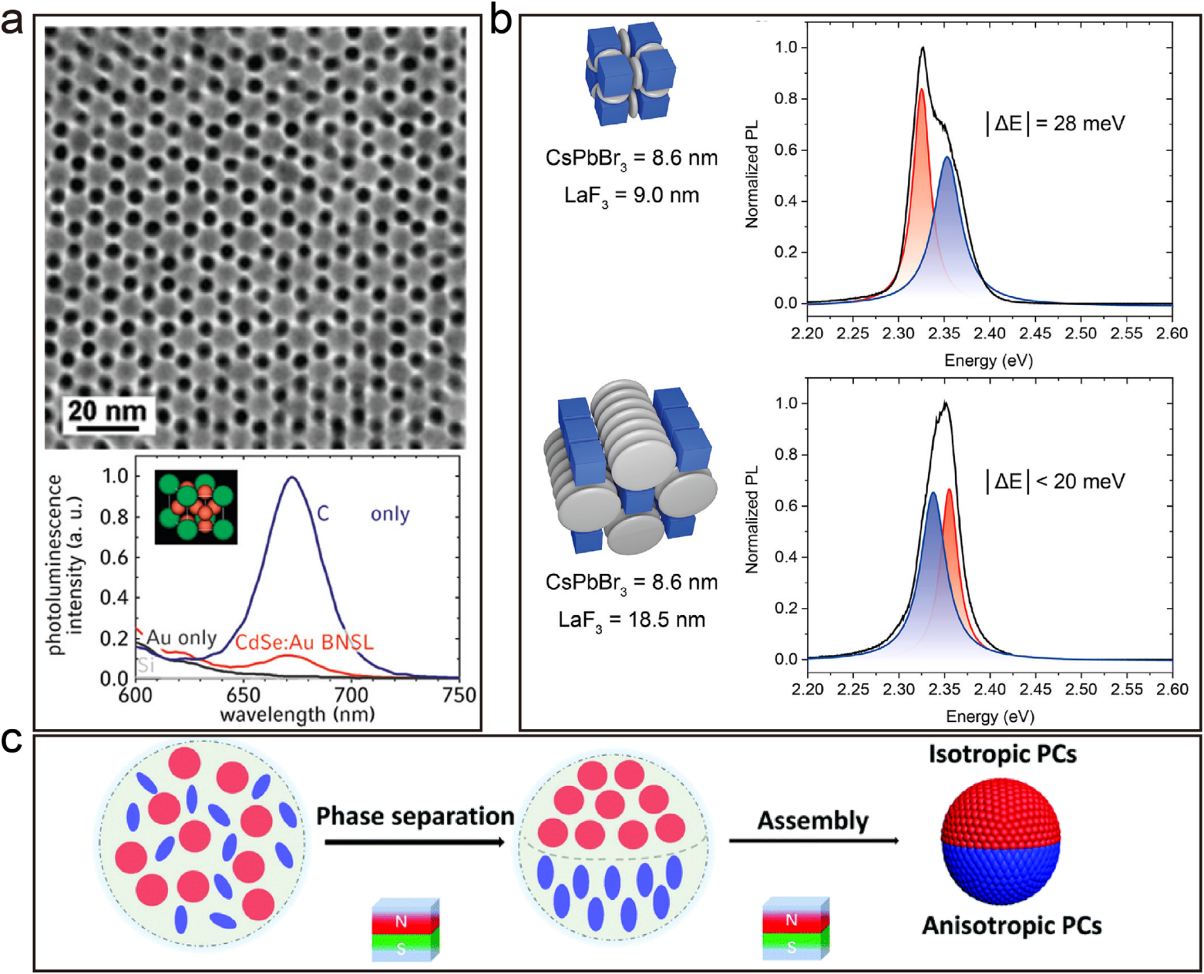
a) TEM images of (001) projections of BNSLs self‐assembled from 8.7 nm CdSe and 5.5 nm Au nanoparticles and fluorescence spectroscopy. Reproduced with permission.^[^ ^31^ ^]^ Copyright 2008, ACS. b) Emission spectrum of ReO_3_‐type SLs and lamellar SLs employing 8.6 nm CsPbBr_3_ nanoparticles and 9 nm LaF_3_ disks. Reproduced with permission.^[^ ^67^ ^]^ Copyright 2021, ACS. c) Schematic illustration showing the formation of Janus PCSs from an aqueous droplet containing nonmagnetic spheres and magnetic ellipsoids. Reproduced with permission.^[^ ^71^ ^]^ Copyright 2021, RSC.

Binary superstructure materials offer limitless opportunities for innovation across various fields due to their unique properties that surpass those of single‐component materials. By selecting nanoparticles with different optical properties for co‐assembly, it is possible to achieve precise modulation of light absorption and emission, generating new optical collective phenomena and expanding their potential applications in a wide range of fields.^[^ ^67^, ^71^ ^]^ For example, the BNLSs consisting of CdS and Au nanoparticles (**Figure** **7**a) establish the conditions to achieve fluorescence quenching of CdS nanoparticles through the coupling between excitons in the CdS nanoparticles and plasmon resonance in the Au nanoparticles. This interaction creates an additional non‐radiative decay channel by transferring resonance energy to the metal nanoparticles.^[^ ^31^ ^]^ Additionally, layered and ReO₃‐type superlattices containing large‐sized 8.6 nm CsPbBr₃ nanoparticles exhibit collective ultrafast photoemission, a phenomenon induced by coherent coupling of the emitting dipoles in the excited state (Figure 7b).^[^ ^67^ ^]^ These properties highlight the potential for tuning collective behaviors through interparticle distances, relative nanoparticle orientations, and interparticle media, such as band charge transport in semiconductor nanoparticle superlattices, dipole interactions in magnetic nanoparticle arrays, or near‐field couplings in plasmonic nanoparticle superlattices. This provides a theoretical and technological foundation for the development of novel optical materials and devices.

The ordering and periodicity of BNLSs offer a wide range of applications in photonic crystallography.^[^ ^67^ ^]^ For instance, a colloidal system consisting of a mixture of nonmagnetic SiO₂ spheres and magnetic Fe₃O₄@SiO₂ ellipsoids (Figure 7c) can be manipulated using a non‐uniform magnetic field to induce phase separation and assist in the subsequent crystallization process.^[^ ^71^ ^]^ This results in the formation of Janus photonic crystal superstructures with distinct regions of isotropic and anisotropic optical properties. Specifically, the non‐magnetic regions exhibit isotropic optical properties, while the magnetic ellipsoidal regions show anisotropic optical properties dependent on the crystal structure. Structural colors can be magnetically tuned by changing the orientation of the hyperparticles. Binary building units, incorporating anisotropic ellipsoids alongside spheres, markedly enhance the structural and functional intricacy of photonic crystals. This combination leads to the development of multifunctional photonic crystals that boast innovative architectures and characteristics. Moreover, such binary systems offer a novel modeling framework for probing the complexities of binary colloidal assembly, thereby advancing our comprehension of these materials' organization principles and potential applications.

#### 3.3.2 Energy Technologies

**Figure 8:**
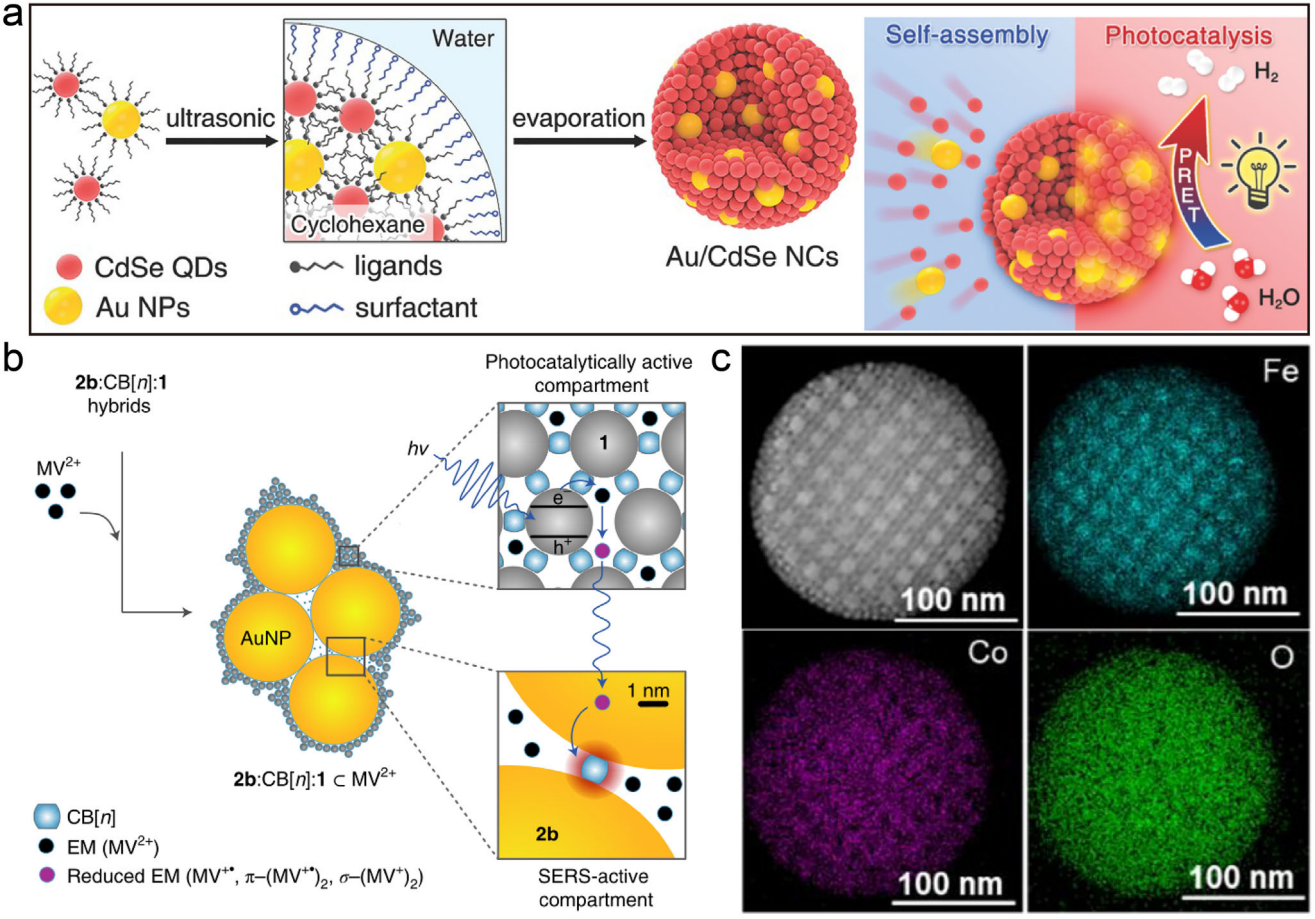
a) Schematic illustration of the self‐assembly process used to synthesize the Au/CdSe NCs. Reproduced with permission.^[^ ^72^ ^]^ Copyright 2017, Wiley‐VCH. b) Schematic of hybrid aggregates emphasizing the role of their compartmentalization and sorption capabilities. Reproduced with permission.^[^ ^26^ ^]^ Copyright 2021, Springer Nature. c) HAADF‐STEM image and the corresponding elemental mapping of AB_13_‐type CoFe_2_O_4_−Fe_3_O_4_ binary superparticles. Reproduced with permission.^[^ ^13^ ^]^ Copyright 2018, ACS.

BNLS technology shows great potential in energy technology, especially in enhancing catalytic performance and improving energy storage performance.^[^ ^13^, ^26^, ^72^ ^]^ By cleverly designing the interactions between different nanomaterials, BNLSs can achieve more efficient electron transfer and increase the reactive sites in the photocatalytic process, which significantly improves the catalytic efficiency. For example, Au/CdSe nanoclusters are formed by self‐assembly, and the plasmonic resonance property of GNPs promotes charge separation on the CdSe quantum dots, which improves the efficiency of photocatalytic hydrogen production (**Figure** **8**a).^[^ ^72^ ^]^ This enhanced effect stems from the plasma‐induced energy transfer mechanism, which enables the generation of additional electron/hole pairs under visible light irradiation, and these high‐energy carriers participate in the reduction reaction, increasing the efficiency of hydrogen production. By adjusting the size and number ratio of nanoparticles as well as the type of surface ligands, the local electric field can be enhanced, which in turn increases the number of electron/hole pairs generated by plasma‐induced generation to optimize the catalytic performance.

In addition, InP/ZnS core‐shell nanoparticles (Figure 8b) and gold nanoparticles can achieve precise assembly control between semiconductor and metal nanoparticles through a self‐limiting assembly mechanism to form colloidally stable hybrid aggregates.^[^ ^26e^ ^]^ These aggregates can efficiently harvest light energy under light conditions and induce non‐equilibrium electron transfer processes, providing the possibility of real‐time monitoring of photogenerated electron transfer. In particular, by combining surface‐enhanced Raman spectroscopy, the researchers were able to directly observe the production of photogenerated radical species and their subsequent kinetic changes, which provides a valuable tool for an in‐depth understanding of complex photochemical reaction mechanisms. The method also provides new ideas for the development of novel and efficient photocatalysts, which can help to advance the development of energy conversion technologies.

Furthermore, the large‐scale preparation of binary superstructure materials provides a new opportunity for the development of high‐performance energy storage materials. Different types of superlattice structures (e.g., AB₁₃, AlB₂, MgZn₂, NaCl, and CaCu₅) can be realized by controlling the ratio of the size to the number of CoFe₂O₄ and Fe₃O₄ nanoparticles (Figure 8c).^[^ ^13a^ ^]^ These BNLSs not only exhibit enhanced magnetic coupling effects but also excellent lithium storage performance, thanks to their characteristic non‐compact stacking arrangement, which helps to maintain structural stability and improve the lithium‐ion diffusion rate during charging and discharging. This tunable assembly method provides an important tool to accelerate the exploration of multicomponent nanocrystalline superlattice materials and heralds the great potential for electrochemical energy storage devices and other high‐tech applications. With the continuous improvement and refinement of their synthesis methods, these materials will play an even more important role in future technological applications.

#### 3.3.3 Biomedical Technology

**Figure 9:**
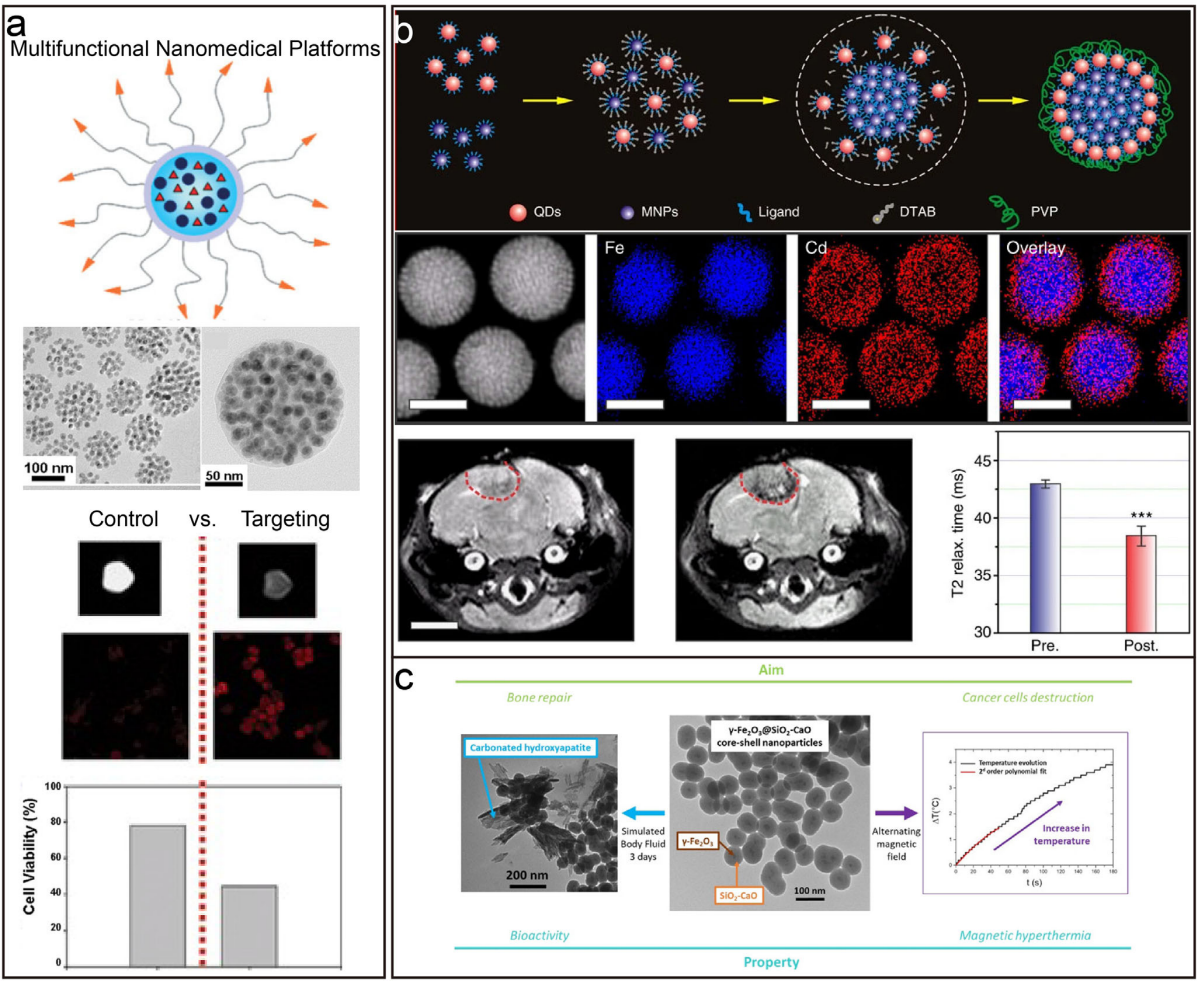
a) A multifunctional polymer nanomedicine platform for both MRI and optical imaging of cancer and effective in vitro drug delivery. Reproduced with permission.^[^ ^73^ ^]^ Copyright 2008, Wiley‐VCH. b) Synthesis, characterizations, and biological applications of core‐shell‐structured SPs. Reproduced with permission.^[^ ^1^ ^]^ Copyright 2014, Springer Nature. c) Superparamagnetic and Bioactive Multicore–Shell Nanoparticles (γ‐Fe_2_O_3_@SiO_2_‐CaO) can repair bone tissue at the same time as being used as a cancer treatment. Reproduced with permission.^[^ ^74^ ^]^ Copyright 2020, ACS.

In modern research at the intersection of nanotechnology and biomedicine, NLSs have great potential for application in bioimaging, drug delivery systems, and biosensor development due to their unique biological activities.^[^ ^1^, ^73^ ^]^ Studies on the bioactivity of BNLSs usually involve the integration of different types of inorganic nanoparticles, such as magnetic iron oxide nanoparticles (Fe₃O₄) or quantum dots (CdSe/ZnS) (**Figure** **9**a), into biodegradable polymer matrices, such as polylactic‐hydroxyglycolic acid copolymer (PLGA).^[^ ^73^ ^]^ This combination not only provides drug carrier functionality but also acts as a magnetic resonance imaging (MRI) contrast agent as well as for optical imaging, enabling deep tissue non‐invasive imaging in living animal models, overcoming the depth limitations of conventional visible light imaging techniques. In addition, Chen et al. have made further advancements in the design of nanomaterials, developing multimodal imaging tools suitable for both magnetic MRI and near‐infrared fluorescence imaging (Figure 9b).^[^ ^1^ ^]^ Their innovative magneto‐fluorescent core‐shell superstructures are self‐assembled from magnetic iron oxide nanoparticles and quantum dots within a polymer matrix. This configuration bestows the super‐nanoparticles with robust magnetic responsivity alongside excellent fluorescence properties. Notably, after surface polyethylene glycosylations, these silica‐coated magneto‐fluorescent super‐nanoparticles can be magnetically manipulated within living cells while maintaining their optical tracking capabilities. Additionally, these materials can serve as in vivo multiphoton and magnetic resonance dual‐modality imaging probes, providing a powerful toolkit for biomedical research. These findings demonstrate the potential of BNLSs in improving diagnostic accuracy and therapeutic efficacy.

Due to their unique physicochemical properties, BNLSs show great potential in the biomedical field to directly intervene in the physiological processes of living organisms.^[^ ^74^ ^]^ For example, BNLSs of magnetic nanoparticles (e.g., Fe₃O₄) and bioactive glasses (e.g., SiO₂‐CaO‐P₂O₅ tethered glass) (Figure 9c) formed by self‐assembly can be guided to the site of injury by using an external magnetic field to promote the local blood circulation and accelerate the tissue repair process.^[^ ^74^ ^]^ Furthermore, Tasar and Ercan's study showed the possibility of combining superparamagnetic iron oxide nanoparticles (SPIONs) with bioactive glass by preparing magnetic bioactive glass nanoparticle composites through two different routes.^[^ ^74^ ^]^ Both approaches successfully yielded magnetic bioactive glass nanoparticles with biocompatible and targeted properties. In in vitro experiments, the presence of SPIONs increased the proliferation rate of osteoblasts in both cases, and in the presence of an external static magnetic field, SDBG nanoparticles specifically increased osteoblast survival, demonstrating the potential for a wide range of biomedical applications, including targeted therapies. Together, these studies demonstrate that nanomaterials with integrated magnetic and bioactive properties can be effectively used in a variety of biomedical fields, including tissue engineering, cancer therapy, and bone regeneration, through rational design and synthesis.

Furthermore, due to its controllable structure, the superlattice can interact with other functional molecules, facilitating the development of multi‐purpose diagnostic and therapeutic platforms.^[^ ^75^ ^]^ For example, encapsulating photosensitizers within vesicles formed by GNPs with strong plasmonic coupling effects can lead to a novel platform capable of three‐modal imaging—near‐infrared fluorescence (NIR), thermal, and photoacoustic imaging—alongside the ability to perform synergistic photothermal and photodynamic therapies.^[^ ^75^ ^]^ By adjusting the size of the vesicles and tuning the position of the Local Surface Plasmon Resonance (LSPR) peak, the light absorption properties are optimized to meet diverse therapeutic needs. Experimental results indicate that under laser irradiation, these superstructures can efficiently penetrate cells, primarily localizing in lysosomes and being internalized via an energy‐dependent endocytosis mechanism. This platform not only enhances therapeutic efficacy but also ensures good biocompatibility and stability, opening new possibilities for biomedical applications, particularly in the diagnosis and treatment of cancer.

Advances in binary assembly research have unveiled fundamental physical‐chemical principles governing self‐organization while demonstrating precision control over nanoscale to microscale architectures through environmental manipulation. Despite progress, conventional methods remain constrained by challenges including unpredictable assembly outcomes, limited scalability, and environmental sensitivity, hindering industrial adaptation, and multifunctional material development. The integration of artificial intelligence is redefining this paradigm. AI‐driven systems employ machine learning to decode assembly behaviors across variable conditions, enabling adaptive control beyond static experimental frameworks. Unlike rule‐based approaches, these platforms utilize real‐time feedback to dynamically optimize assembly pathways, fostering structurally intricate and functionally tailored architectures. This synergy between AI and assembly science amplifies precision in nanoscale engineering while accelerating the creation of next‐generation smart materials and devices.

## 4 AI‐Guided Programmable Assembly of Colloidal Nanoparticles

The current binary assembly of colloidal nanoparticles is mainly based on the concept of passive design, which assembles two different types of nanoparticles through natural interactions such as electrostatic forces, van der Waals forces, etc. Although this method has been successful in some applications, with the advancement of technology and the increasing demand for applications, traditional passive assembly methods are no longer able to meet the increasingly complex and diverse practical needs. Based on the substrate selectivity and arrangement diversity of BNLSs, we can foresee that an important future trend in this field will be the realization of on‐demand fabrication of highly complex materials by AI‐guided programmable assembly.^[^ ^76^ ^]^ We envisage that in the future, it will be possible, similar to writing a computer software programme, the rules for combining nanocrystals will be precisely designed by a variety of advanced technological means. In this way, nanostructures can be carefully programmed and positioned to achieve specific arrangements and functions, just like writing computer code. We envisage that in the future, it will be possible, similar to writing a computer software programme, the rules for combining nanocrystals are precisely designed by a variety of advanced technological means. In this way, nanostructures can be carefully programmed and positioned to achieve specific arrangements and functions, just like writing computer code. The term ‘AI‐guided programmable assembly’ here differs from traditional computer programming. It refers to controlling the assembly and precise positioning of nanocrystals through various technological means, guided by the desired structure and properties of the target nanomaterials. This approach allows for precise manipulation and customization using advanced technologies and AI techniques to ensure that the generated material meets specific requirements.

AI‐guided programmable assembly aims to reduce common disorders and structural defects in passive assembly through enhanced control over functionality, position, and arrangement of nanoparticles. Through precise control of the assembly process, binary assembly ensures high consistency and efficiency in large‐scale production, reducing batch‐to‐batch variations. AI‐guided binary assembly can achieve flexibility and diversity in material combinations. By encoding the functionality of each nanoparticle, researchers can flexibly select particle types, assembly methods, and arrangement sequences according to needs, allowing different functional modules to be combined as required, thereby creating new multifunctional materials. In addition, the AI‐guided binary assembly gives a greater opportunity to endow nanomaterials with ‘smart’ properties, enabling them to self‐regulate in response to changes in the external environment, thus becoming a smart material capable of responding to external stimuli and performing specific tasks.

The ability to control the arrangement and composition of nanoparticles opens up new avenues for designing materials with highly tunable properties. For example, by precisely controlling the spacing between nanoparticles, it is possible to modulate their electronic, optical, or catalytic properties to meet specific needs. This programmability also enables the design of dynamic materials that can adapt to environmental changes or stimuli, such as light, heat, or chemical signals. These materials could find applications in fields ranging from sensors and energy storage to drug delivery and environmental monitoring. As the technology continues to mature, the integration of AI‐guided programmable assembly with other nanotechnologies could lead to the creation of multifunctional materials with unprecedented capabilities, further bridging the gap between fundamental research and real‐world applications.

### 4.1 AI‐Guided Programmable Assembly Research Basis

Advancements in mono‐ and binary assembly techniques for colloidal nanoparticles have established a robust foundation for exploring more complex, AI‐guided programmable assembly. By investigating these fundamental assembly methods, researchers have significantly enhanced their understanding of the physicochemical interactions among nanoparticles, including van der Waals forces and electrostatic interactions. This accumulated knowledge enables scientists to guide nanoparticles into self‐assembling according to predefined patterns, leveraging various physicochemical forces to create innovative functional materials. These materials range from efficient photoelectric conversion materials and highly sensitive sensors to advanced catalysts and cutting‐edge photonic devices. With the rapid development of artificial intelligence (AI) technology, its unique advantages in resolving complex multi‐scale interactions, predicting dynamic assembly behaviors, and optimizing material properties have injected a revolutionary momentum into nanoscience. Such progress not only drives the field of nanotechnology toward greater practical utility but also encapsulates a systematic assembly approach. This methodical process consists of four critical stages, each essential for developing nanomaterials with specific structures and functionalities. Through the disciplined execution of these stages, scientists ensure that the final assemblies possess the desired characteristics and performance tailored for targeted applications, thereby propelling nanotechnology into new realms of possibility and application.

#### 4.1.1 Advances in Nanoparticle Synthesis Technology

The initial step in the process of AI‐guided programmable assembly of colloidal nanoparticles involves material synthesis, which is essential to ultimately obtain nanomaterials with specific structures and functions. With the continuous advancement of nanomaterial synthesis techniques, researchers are now able to achieve highly precise control over the size and morphology of nanoparticles. Through precise manipulation of reaction conditions, nanoparticles with diverse morphologies—ranging from spherical and rod‐like to sheet‐like and even polyhedral—can be synthesized. Concurrently, advancements in synthesis technology have enabled the fabrication of multi‐component nanoparticles, encompassing alloy nanoparticles, core‐shell nanoparticles, heterojunctions, and other intricate structures. These multi‐component nanoparticles exhibit remarkable potential for application owing to their distinctive physicochemical properties.^[^ ^1^, ^4^, ^77^ ^]^ However, conventional nanoparticle synthesis relies heavily on manual processes based on intuition and trial‐and‐error methods, which typically require a significant amount of time and resources to explore the vast synthesis parameter space. To overcome these challenges, researchers have begun to adopt machine learning (ML) methods for a data‐driven approach to material discovery. ML can analyze vast datasets derived from experimental results and theoretical models to predict optimal conditions for synthesizing nanoparticles with desired properties. Machine learning models can be trained on historical data to identify patterns that are not immediately apparent to human researchers, leading to the discovery of new synthesis pathways or optimization of existing ones. AI‐driven systems can also perform real‐time monitoring and adjustment during the synthesis process, ensuring consistent quality and performance of the nanoparticles produced.

**Figure 10:**
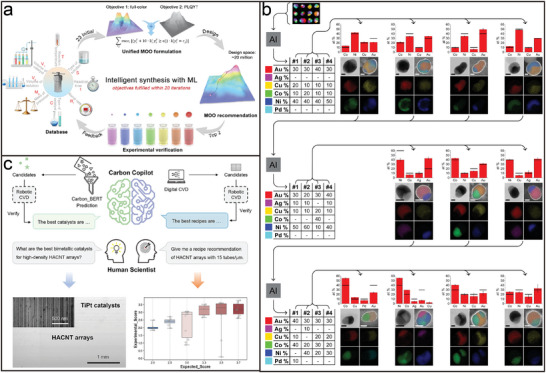
a) Workflow of ML‐guided synthesis of carbon quantum dots (CQDs) with superior optical properties. Reproduced with permission.^[^ ^78^ ^]^ Copyright 2024, Springer Nature. b) Closed‐loop optimization for the discovery of quaternary metallic SINPs. Reproduced with permission.^[^ ^79^ ^]^ Copyright 2021, AAAS. c) Schematic diagram of the artificial intelligence‐driven Carbon Copilot (CARCO) platform. Reproduced with permission.^[^ ^80^ ^]^ Copyright 2025, Cell.

In recent years, research in ML‐assisted nanoparticle synthesis has grown rapidly, providing new avenues for accelerated material discovery and optimization.^[^ ^78^, ^81^ ^]^ This field combines advanced experimental techniques with computational science, aiming to explore nanoparticle synthesis conditions and optimize performance through data‐driven approaches. For example, Guo et al.^[^ ^78^ ^]^ proposed a multi‐objective optimization strategy to guide the hydrothermal synthesis of carbon quantum dots (CQDs) using gradient‐boosting decision tree models such as XGBoost (**Figure** **10**a). They achieved a high quantum yield (more than 60%) of panchromatic fluorescent CQDs in only 63 experiments, demonstrating that ML‐guided experiments can drastically shorten the research cycle and outperform conventional methods. On the other hand, Bayesian optimization, as an effective global optimization algorithm, has been applied to the synthesis of many types of nanomaterials. Wahl et al.^[^ ^79^ ^]^ explored and successfully synthesized 18 never‐before‐seen complex heterojunction nanomaterials (Figure 10b) in 8D chemical space (including gold, silver, copper, cobalt, nickel, palladium, tin, and platinum) by using the Bayesian optimization algorithm to guide experimental processes, including extremely complex biphasic nanoparticles. In addition, high‐throughput synthesis of organic semiconductors was combined to create large datasets, and Bayesian optimization was used to discover novel hole‐transporting materials with tailored properties for solar cell applications. Predictive models are based on molecular descriptors, making it possible to relate the structure of these materials to their properties and achieve photovoltaic conversion efficiencies of up to 26.2% in chalcogenide solar cells.^[^ ^81^ ^]^ These researches demonstrate how machine learning‐driven materials discovery can accelerate the development of complex nanomaterials and have the potential to change the way materials are explored across multiple applications.

In addition, the development of automated experimental workstations is critical to improving the efficiency of nanoparticle synthesis. Such platforms are capable of automating a range of operations, reducing human error, and speeding up experiments. Li et al.^[^ ^80^ ^]^ developed an AI‐driven platform called CARCO (Figure 10c), specifically targeting CBNs, specifically horizontally aligned carbon nanotube (HACNT) arrays. The platform identified a breakthrough titanium–platinum bimetallic catalyst through high‐throughput screening of catalysts, surpassing iron catalysts, which have been considered the best catalysts for growing high‐density HACNT arrays since the 2000s. CARCO has also achieved controllability of the growth density of HACNT arrays with the help of virtual experiments, which greatly enhances customization capabilities. Combined with an efficient autonomous flow chemistry system, real‐time Bayesian optimization algorithms, and other means allows the production of high‐quality nanomaterials at multiple target peaks, enabling fully autonomous materials chemistry exploration while improving experimental efficiency and material quality. As the research progresses and the technology matures, it is expected that ML will play an increasingly important role in the field of nanoparticle synthesis, facilitating the discovery and application of more high‐performance materials and providing the necessary conditions for the AI‐guided assembly of nanoparticles.

#### 4.1.2 Development of Surface Modification and Functionalization Technologies with Fine Regulation of Nanoparticle Interactions

**Figure 11:**
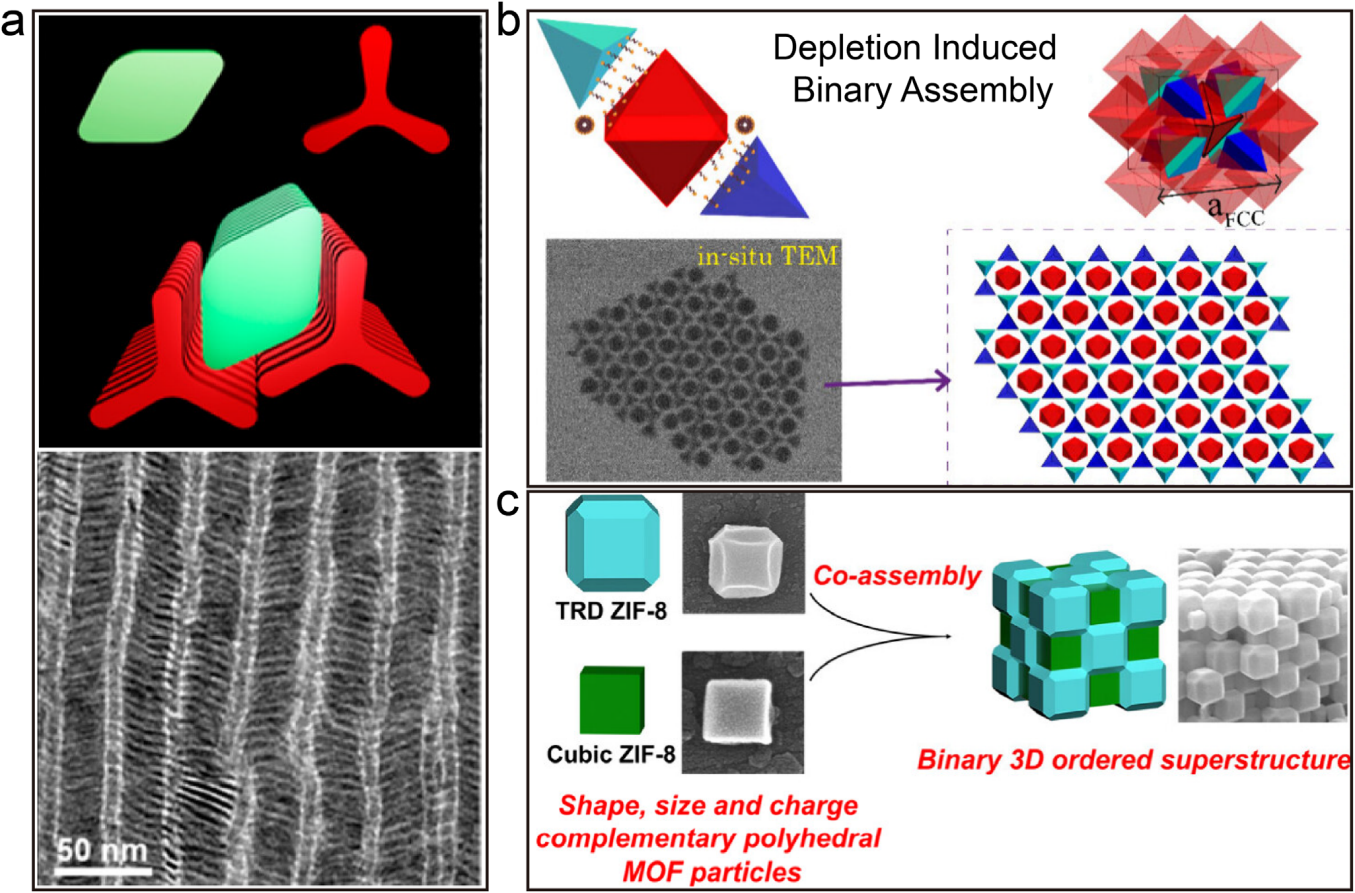
a) Schematic diagram of binary assembly and TEM image of binary self‐assembly formed by the interaction of tripodal nanoplates and rhombic nanoplates through complementary shapes. Reproduced with permission.^[^ ^11^ ^]^ Copyright 2013, ACS. b) Schematic and in situ TEM of depletion‐induced tunable assembly of complementary platonic solids. Reproduced with permission.^[^ ^82^ ^]^ Copyright 2024, ACS. c) 3D NaCl‐type binary porous superstructures formed by the co‐assembly of two colloidal polyhedral metal‐organic framework (MOF) particles with complementary sizes, shapes, and charges. Reproduced with permission.^[^ ^69^ ^]^ Copyright 2024, ACS.

After material synthesis, the second stage in colloidal nanoparticle AI‐guided programmable assembly involves the precise identification and design of the assembly process. This stage is centered on shape recognition^[^ ^11^ ^]^ and molecular recognition^[^ ^82^ ^]^ to ensure that the nanoparticles interact in a predetermined way and assemble into complex structures (**Figure** **11**a,b). This process not only relies on an in‐depth understanding of the physicochemical properties of the nanoparticles but also demands precise design and control. Shape recognition entails leveraging the unique geometrical morphology of nanoparticles to steer their directed assembly. By controlling the synthesis conditions to fabricate nanoparticles with specific shapes, these shape properties can guide the self‐assembly process. Shape recognition is pivotal for constructing complex ordered superstructures, allowing nanoparticles to organize themselves into desired patterns without external intervention. On the other hand, molecular recognition involves utilizing specific interactions between particular molecules or molecular groups to achieve selective binding of nanoparticles. This recognition mechanism can be based on various interactions such as hydrogen bonding, metal‐ligand coordination, π‐π stacking, and hydrophobic effects, among others. For example, in the process of A recognizing B, one nanoparticle (A) is modified with a specific functional molecule or ligand on its surface that can selectively recognize and bind to a complementary molecule on another nanoparticle (B). Through this approach, A and B can serve as fundamental units (AB motifs) for more intricate assemblies.^[^ ^19^, ^83^ ^]^

To date, significant progress has been achieved in the field of nanomaterials regarding the precise identification of materials.^[^ ^11^, ^84^ ^]^ Researchers are currently harnessing advanced synthesis techniques and surface modifications to achieve precise recognition of nanoparticles. The progress in molecular recognition technology has empowered nanoparticles to incorporate specific functional groups or ligands on their surfaces for targeted binding. Mechanisms like DNA hybridization, antibody‐antigen interactions, and metal‐ligand coordination have been effectively utilized for selective assembly between nanoparticles. Furthermore, they are employing multiple physical property detection methods to enable real‐time monitoring and feedback control, thereby advancing the field of nanomaterials (Figure 11c).^[^ ^69^, ^85^ ^]^ To enhance the precision and reliability of recognition, scientists are exploring multimodal recognition strategies by amalgamating shape recognition and molecular recognition to boost specificity.

**Figure 12:**
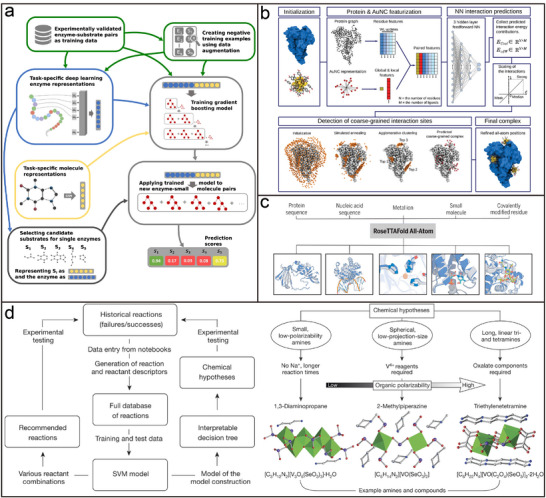
a) ESP model validation enzyme‐substrate relationship diagram. Reproduced with permission.^[^ ^87^ ^]^ Copyright 2023, Springer Nature. b) Schematic diagram of the GraphBNC method combining graph theory and neural networks to predict atomic‐level interactions between metal nanoclusters and proteins. Reproduced with permission.^[^ ^87^ ^]^ Copyright 2024, Wiley‐VCH. c) Schematic representation of RoseTTAFold All‐Atom predictions of biomolecular assemblies, including proteins, nucleic acids, metals, small molecules, and covalent modifications. Reproduced with permission.^[^ ^88^ ^]^ Copyright 2024, AAAS. d) Schematic representation of the feedback mechanism in the dark reactions project and the graphical representation of the three hypotheses generated from the model, and representative structures for each hypothesis. Reproduced with permission.^[^ ^89^ ^]^ Copyright 2016, Springer Nature.

To further enhance the efficiency and accuracy of this process, the application of AI opens up entirely new possibilities.^[^ ^86^ ^]^ Through the use of machine learning algorithms and deep learning models, AI can simulate and predict complex interactions between molecules, thereby assisting scientists in sifting through the vast number of candidate molecules to find the most suitable functional molecule or ligand.^[^ ^87^ ^]^ This capability not only accelerates the research process but also increases the success rate of finding molecules with desirable properties. Specifically, in the field of enzyme‐substrate relationship research (Figure 12a),^[^ ^87^ ^]^ the ESP model can predict different enzyme‐substrate pairs with more than 91% accuracy for a wide range of enzymes and metabolites, and the model uses a modified Transformer architecture to represent enzymes, which is trained by introducing randomly sampled small molecules as non‐substrates through a data augmentation method that allows it to work efficiently even when there is a lack of information about non‐substrates. Meanwhile, the GraphBNC method^[^ ^87^ ^]^ demonstrates how to combine graph theory and neural networks to predict atomic‐scale interactions between metal nanoclusters and proteins without the need for pre‐existing experimental information (**Figure** **12**b), which provides strong support for applications such as bioimaging, biosensing, and nanomedicine. In addition, models such as RoseTTAFold All‐Atom and AlphaFold3 (Figure 12c) demonstrate the possibility of predicting general intermolecular interactions using existing structural data.^[^ ^88^ ^]^ These models extend neural network architectures to handle diverse biomolecules and employ large‐scale protein structure databases to predict protein structures and their binding conformations with other molecules. Their ability to capture interactions between different types of molecules represents an important step toward the development of AI systems that can provide a comprehensive understanding of the molecular basis of life. Together, these advances show the great potential of AI in computational molecular science, particularly in the modeling of non‐bonded interactions.

Further, AI can assist in the design and synthesis of organic molecular ligands to ensure that the best functional molecules for nanoparticle surface modification are achieved. Machine learning models can take into account a variety of factors such as reaction conditions, yields, cost‐effectiveness, and environmental impact to ensure that the end product is both efficient and sustainable.^[^ ^90^ ^]^ Specifically, machine learning methods have been used to predict and optimize chemical reaction pathways, select appropriate reaction conditions, and identify compounds that are most likely to succeed. A database of chemosynthetic reactions based on experimental results, combined with support vector machine (SVM) and decision tree algorithms, was trained and tested on 3955 complete sets of hydrothermal synthesis reaction data to predict the crystallization of alum selenite.^[^ ^89^ ^]^ Its prediction accuracy was 89%, which is even more accurate than manual synthesis by experienced chemists. In addition, a graphical representation was developed to show three hypotheses generated from the model, representing different amines and their corresponding compounds, to guide the selection and synthesis of new compounds (Figure 12d). Therefore, AI‐assisted surface modification and functionalization technology can significantly improve our understanding, design, and control of the nanoparticle self‐assembly process, and promote the development of the nanotechnology field to a new level.

#### 4.1.3 Simulation Computing Techniques Predict Evolving Assembly Structures

In terms of simulation methods underlying assembly prediction, advances in computational modeling have established simulation techniques as pivotal tools for decoding multiscale self‐assembly mechanisms. Glotzer's group pioneered entropy‐driven assembly design using shape‐engineered patchy particles, employing Monte Carlo simulations to elucidate how anisotropic interactions govern crystalline and quasicrystalline ordering. ^[^ ^10^, ^17^, ^91^ ^]^ Their work extended to inverse design strategies, identifying over 350 colloidal self‐assembly templates for 3D photonic crystals with tailored bandgap properties.^[^ ^92^ ^]^ Complementing this, Widmer‐Cooper provides insight into the surfactant distribution of nanorods through the use of dissipative particle dynamics simulations.^[^ ^93^ ^]^ In addition, Escobedo's team has achieved several advances. First, their Monte Carlo simulations of the intermediate phase behavior of polyhedral particles have led to the formulation of prediction criteria and highlighted the importance of kinetic disorder.^[^ ^94^ ^]^ Second, they investigated the disorder‐to‐order phase transition in hard cube systems, measured interfacial free energy, and explored the effects of low interfacial tension on these phenomena.^[^ ^95^ ^]^ Travesset's molecular theory (MOLT‐CF) further decoded nanocrystal assembly pathways, demonstrating enthalpy‐dominated bcc and entropy‐driven fcc lattice formation through competitive thermodynamic landscapes.^[^ ^96^ ^]^

The synergy of inverse design frameworks with machine learning has revolutionized predictive accuracy in colloidal architectures. Torquato's hybrid model, combining density functional theory with neural networks, enabled intelligent screening of optimal interaction potentials for target colloidal configurations.^[^ ^97^ ^]^ In the context of self‐assembly, Torquato's team used inverse analysis approaches to find interaction potentials for targeted crystal and disordered ground states, which helps in understanding the relationship between collective structural behavior and interactions.^[^ ^98^ ^]^ Dijkstra's team advanced this paradigm by introducing a generic inverse design method that combines an evolutionary strategy for parameter optimization and a convolutional neural network as an order parameter to reverse‐engineer crystals, quasicrystals, and liquid crystals by targeting their diffraction patterns.^[^ ^99^ ^]^ This method can optimize colloidal interactions and thermodynamic conditions for the self‐assembly of a target phase, and has demonstrated robustness and versatility in various model systems, including 2D and 3D cases. The team also combined machine learning‐assisted coarse‐grained models with Monte Carlo simulations, successfully predicting non‐spherical particle interactions and elucidating the transition from linear arrangements to 3D clusters in confined spherical systems, thus offering novel insights for materials design.^[^ ^100^ ^]^ Grunwald's team focuses on nanocrystalline superlattice self‐assembly and organic molecule co‐crystallization, and provides a theoretical basis and guidance for precise inverse design of materials through computational simulation to investigate the processes and analyze the influencing factors.^[^ ^6^, ^101^ ^]^

**Figure 13:**
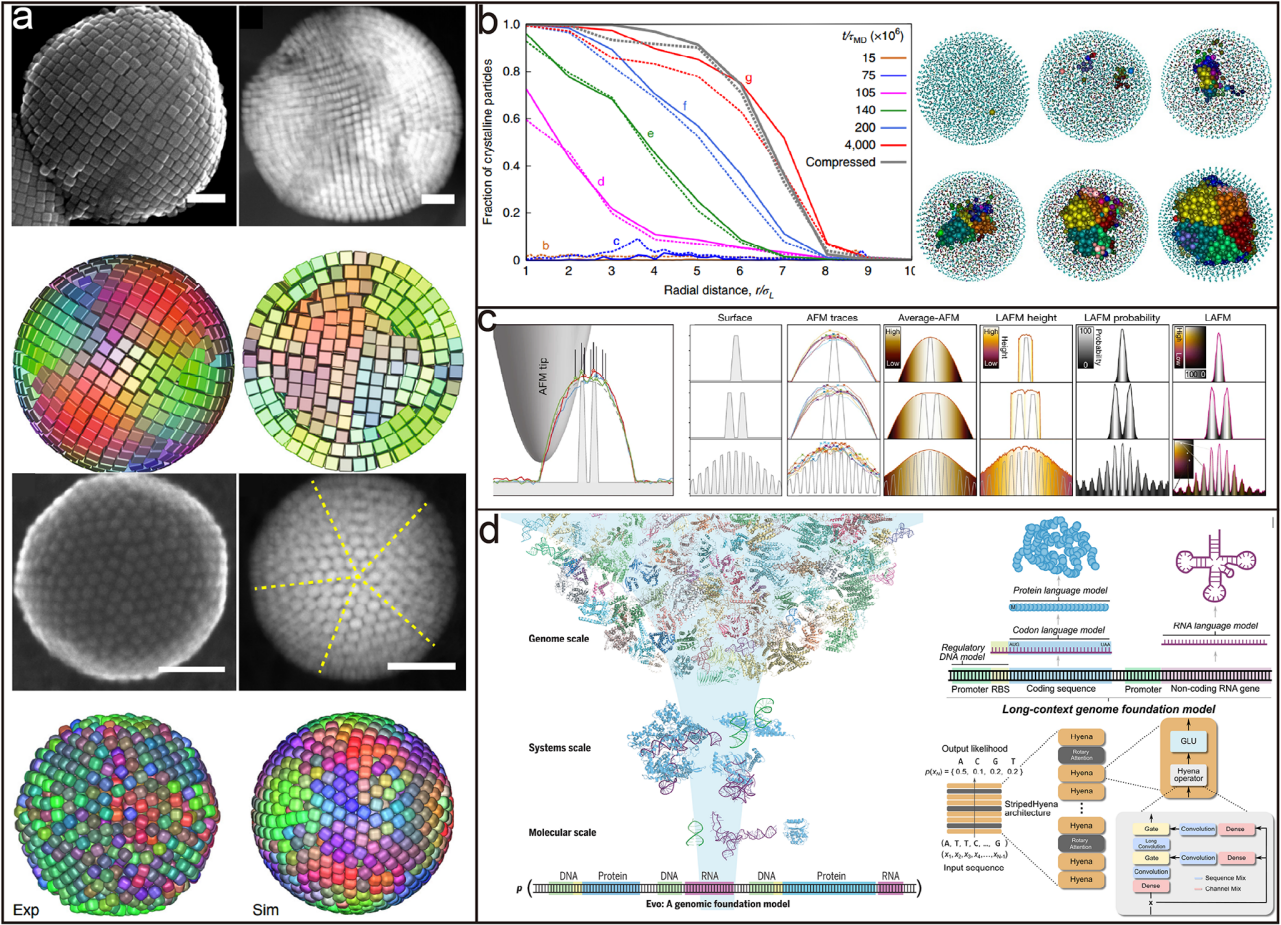
a) TEM characterisation of the assemblies of sharp nanocubes and round nanocubes, with computer simulations. Reproduced with permission.^[^ ^102^ ^]^ Copyright 2018, Springer Nature. b) Nucleation and growth of the binary icosahedral cluster in simulations. Reproduced with permission.^[^ ^35^ ^]^ Copyright 2021, Springer Nature. c) Schematic of an AFM tip scanning a high topography with high‐resolution features. Reproduced with permission.^[^ ^107^ ^]^ Copyright 2021, Springer Nature. d) Evo foundation model schematic and Pretraining a genomic foundation model across prokaryotic life. Reproduced with permission.^[^ ^108^ ^]^ Copyright 2024, AAAS.

The emergence of experimentally validated multiscale simulations has made it possible to analyze the dynamics of constraint‐driven assemblies. A paradigm‐shifting contribution by Wang et al. exemplifies this progress, where multiscale simulations coupled with experimental validation elucidated the synergistic interplay between spherical confinement and particle geometry in directing assembly pathways.^[^ ^35^, ^102^ ^]^ By combining multiscale simulations with single‐particle‐resolution electron tomography, decoding geometric confinement effects: Monte Carlo simulations of rounded cubes revealed critical thresholds for cubic‐to‐icosahedral transitions (**Figure** **13**a), validated by experimental observation of competing local ordering and global confinement.^[^ ^102^ ^]^ This “simulate‐predict‐validate” methodology transcends conventional trial‐and‐error approaches, establishing a robust framework for rational superlattice engineering. Subsequent molecular dynamics investigations of binary hard‐sphere systems further revealed eccentric nucleation mechanisms in icosahedral clusters (Figure 13b),^[^ ^35^ ^]^ constrained by interfacial lamellar fluid phases—a pivotal advancement for understanding confined multicomponent co‐assembly. Extending this framework to anisotropic systems, their study of nanoplatelets combined molecular dynamics simulations with quantitative 3D orientation analysis via electron tomography.^[^ ^35^ ^]^ For disk‐shaped nanoparticles, simulations predicted a structural transition threshold, and for triangular and leaf‐shaped nanoplatelets, simulations successfully predicted confinement‐induced symmetry breaking and twisted columnar architectures, later confirmed by tomography. These findings established a multiparameter model linking shape anisotropy, spatial constraints, and interaction potentials, enabling AI‐accelerated superlattice design.

Recent methodological syntheses highlight the necessity of multiscale approaches for functional material design. Bassani et al. emphasized integrating density functional theory (DFT) with coarse‐grained models and kinetic Monte Carlo (KMC) methods to correlate nanostructure‐function relationships.^[^ ^31^ ^]^ Haji‐Akbari and his team have provided insights into the distribution of surfactants in gold nanorods by developing and applying a variety of computational models and simulation methods, such as molecular Dynamics (MD) simulations, coarse‐grained models, and Markov state models etc., to study self‐assembly and molecular organization in depth.^[^ ^103^ ^]^ Despite these advances, challenges persist in predicting non‐equilibrium assembly trajectories, urging the development of real‐time adaptive simulation platforms. Current trends suggest a paradigm shift from static structure prediction to dynamic process engineering, with machine learning‐potential hybrid models emerging as key tools for next‐generation programmable materials.

#### 4.1.4 Continuing Maturation of External Regulatory Instruments

The final stage involves assembly prediction and device manipulation, which is key to turning the design into reality. Transitioning from theoretical models to physical realization requires precise environmental control and advanced manipulation technologies. During this phase, external conditions such as temperature, humidity, electric fields, and magnetic fields suitable for nanoparticle assembly are established and maintained to ensure the stability and controllability of the entire assembly process. Automated equipment and precision manipulation techniques like microfluidic systems, robotic arms, or specialized assembly platforms are employed to execute the predetermined assembly procedures. The assembly process is monitored closely, allowing for timely adjustments based on real‐time feedback to achieve optimal results.

Notably, the technologies for manipulating single particles, specific types of nanoparticles, and multiple types of nanoparticles simultaneously have reached a considerable level of maturity.^[^ ^104^ ^]^ For single‐particle manipulation, tools like optical tweezers and atomic force microscopy (AFM) tips can achieve high‐precision positioning with accuracies down to tens of nanometers or even smaller. In the case of manipulating a single class of nanoparticles, surface chemical modifications or the use of external fields (like electric or magnetic fields) enable highly controlled arrangement and assembly. DNA nanotechnology stands out as an exceptional method for AI‐guided self‐assembly, allowing precise nanostructure assembly through the design of DNA sequences.^[^ ^105^ ^]^ This approach not only offers high controllability and biocompatibility but also enhances the capability to create complex nanoscale architectures. Simultaneous manipulation of multiple types of nanoparticles has also seen significant advancements. By integrating microfluidic systems, robotic arms, or specialized assembly platforms, researchers can manipulate different types of nanoparticles on a microscopic scale to construct complex structures. Particularly, the combination of optical tweezers with electric or magnetic fields enables the precise control of multiple nanoparticles at once, playing a pivotal role in research on AI‐guided self‐assembly.^[^ ^106^ ^]^ This integration plays a pivotal role in research into AI‐guided self‐assembly.

AI has become an integral part of the vast field of assembly technology, significantly improving the efficiency and precision of machine operations and assembly processes. It is a key bridge between theory and practice, where abstract concepts become accessible through concrete implementation. Advanced technologies such as optical tweezers, microfluidic systems, robotic arms, and atomic force microscopes rely on AI to optimize the operation of automated machinery and achieve unprecedented positioning accuracy. For example, researchers have developed Localization Atomic Force Microscopy (LAFM) (Figure 13c) using algorithms combined with fluorescence localization microscopy to apply localization algorithms to the analysis of spatial fluctuations of topographical features in AFM and High Speed Atomic Force Microscopy (HS‐AFM) images.^[^ ^107^ ^]^ This approach reveals high‐resolution images of protein surface details at the angstrom level of detail. This not only enhances the control of details in the production process but also reduces the incidence of human error, ensuring the stability and reliability of product quality. In the field of DNA nanotechnology, software tools powered by AI are revolutionizing the way complex DNA sequences are designed, paving the way for the construction of highly controllable and biocompatible nanostructures. A cutting‐edge study introduces a genome‐based macromodel called Evo (Figure 13d).^[^ ^108^ ^]^ Using the latest advances in deep signal processing, the Evo model was extended to include seven billion parameters and is capable of handling context lengths of up to 131 kilobases at single‐nucleotide resolution. Evo's powerful ability to predict, generate, and design entire genome sequences not only promises to revolutionize the paradigm of synthetic biology but also offers unprecedented possibilities for the fabrication of complex nanostructures. These technical tools complement each other and together facilitate the precise design and manipulation of AI‐guided programmable assembly.

### 4.2 Outlook for AI‐Guided Programmable Assembly

#### 4.2.1 Technical Prospect of AI‐Guided Programmable Assembly

**Figure 14:**
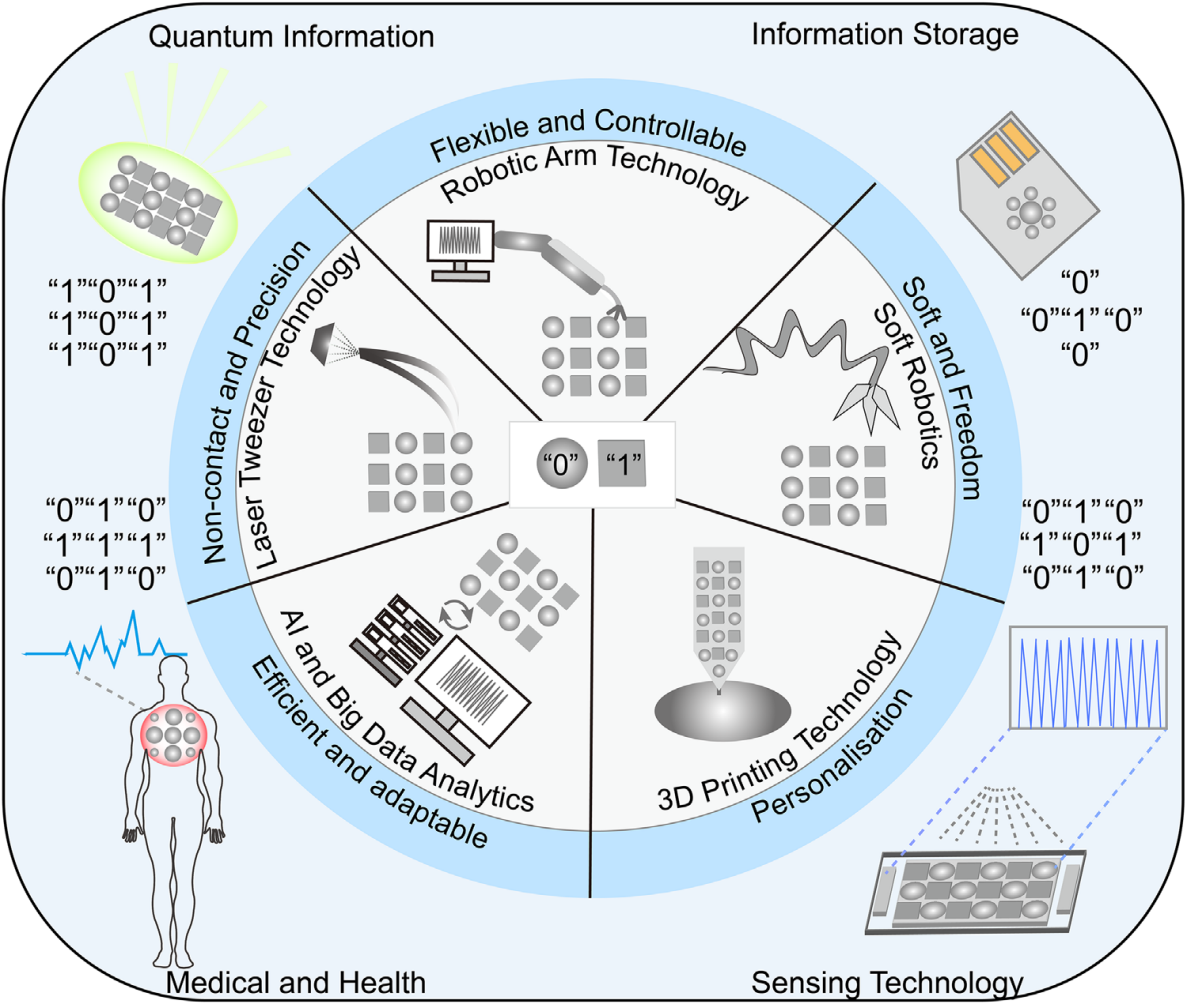
Summary diagram of the preamble technology for AI‐guided assemblies.

With the swift advancement of technology, a series of cutting‐edge technologies is expected to gradually change the future of AI‐guided programmable assembly (**Figure** **14**). The widespread use of robotic arms and soft robots can not only significantly enhance the flexibility and adaptability of AI‐guided programmable assembly, but also significantly improve production efficiency and precision.^[^ ^109^ ^]^ The introduction of laser tweezer technology can make the precision assembly of tiny parts possible, especially in the electronics and biomedical fields, and the progress of 3D printing technology can open up a new mode of on‐demand manufacturing and customized production, significantly reducing the cost and time of prototyping and small batch production. At the same time, the integration of AI technology is expected to bring intelligent decision support and optimization capabilities to AI‐guided programmable assembly. Through data analysis and machine learning, self‐adjustment and optimization of the production process can be achieved, further enhancing the reliability and responsiveness of the system. The integrated application of these technologies will not only promote the transformation of manufacturing to a higher level of automation and intelligence but also promote the sustainable development of the industry and open up a broader application prospect for AI‐guided programmable assembly technology.

##### Robotic Arm Technology in AI‐Guided Programmable Assembly

**Figure 15:**
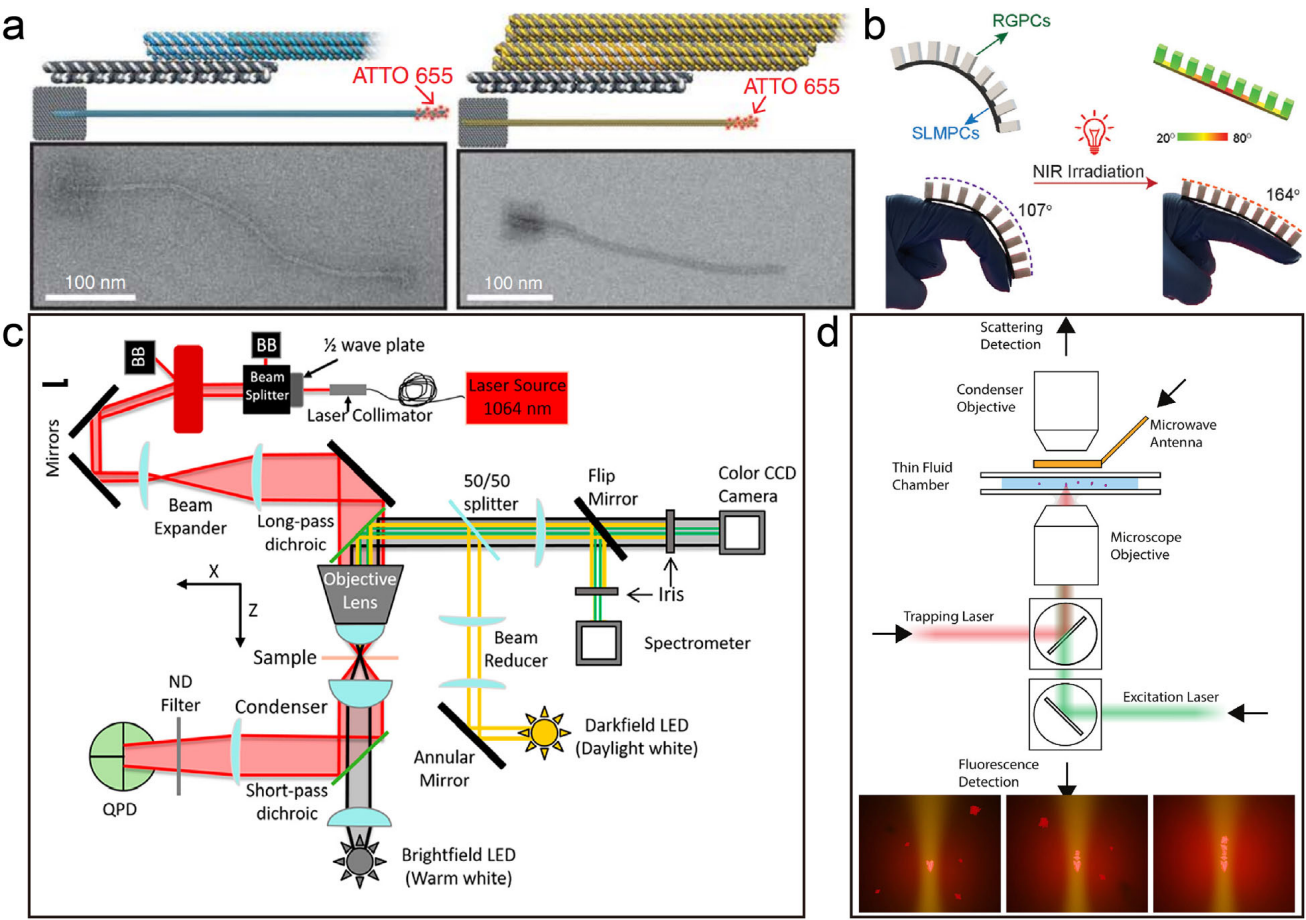
a) External electric control of the robotic arm, two pointer extension designs for the robot arm, and corresponding TEM images. Reproduced with permission.^[^ ^111^ ^]^ Copyright 2018, AAAS. b) The schematic diagram and demonstration of HGNPCs as potential external wearable assistive applications. Reproduced with permission.^[^ ^114^ ^]^ Copyright 2024, Wiley‐VCH. c) Instrument schematic of the custom‐built optical tweezer and DF microscope. Reproduced with permission.^[^ ^117^ ^]^ Copyright 2023, ACS. d) Schematic showing the main elements of the experimental apparatus and the illustration of the nanodiamond self‐assembly process, where the particles assemble at the focus of infrared optical tweezers. Reproduced with permission.^[^ ^116^ ^]^ Copyright 2024, ACS.

The integration of robotic arm technology into the field of AI‐guided programmable assembly of nanomaterials is expected to have a transformative impact on this area of expertise.^[^ ^110^ ^]^ According to Kopperger et al.,^[^ ^111^ ^]^ a self‐assembling DNA nanorobotic arm controlled by an electric field demonstrates superior high‐precision positioning and stable handling performance (**Figure** **15**a), capable of performing material handling, placement, and assembly tasks at the nanoscale with unrivaled precision and reliability. For example, the nanorobot arm can respond to external electric field commands in millisecond time, achieve extensions of up to 400 nm on a 55 nm x 55 nm platform, and precisely switch between arbitrary positions on the platform. It is also capable of applying pico‐Newtonian‐level forces for electrically driven transport of molecules or nanoparticles, as well as demonstrating its manipulation capabilities through force‐induced DNA double‐strand melting experiments.

The technological advancements mentioned enable the precise fabrication of intricate nanostructures, such as arrays of nanowires, nanotubes, and nanoparticles, by ensuring the accurate alignment and orientation of individual nanoelements. The integration of robotic arms in fabrication processes facilitates the adoption of advanced techniques for nanostructure fabrication and surface modification, leading to heightened productivity, enhanced product quality, and reduced manufacturing costs. This technological foundation supports both the research and development (R&D) and industrialization of nanotechnology, fostering innovation in materials science and advancements in micro‐ and nano‐fabrication processes. The application of robotic arm technology in nanomaterial assembly marks a significant breakthrough in nanotechnology. For instance, this technology can be upscaled to larger hybrid systems by combining lithography and self‐assembly methods to create filamentary or multi‐armed structures. It may even lead to the development of algorithmic self‐assembly processes to generate diverse robotic platforms tailored for specific tasks. These advancements extend beyond academic realms, presenting novel opportunities for industrial applications in fields like biomedicine, information technology, and environmental sciences. The integration of robotic arm technology in nanotechnology not only revolutionizes the way nanostructures are fabricated but also paves the way for innovative applications across various industries, showcasing the transformative potential of nanotechnology in diverse sectors.

##### Soft Robotics in AI‐Guided Programmable Assembly

The advent of soft robotics marks a significant milestone in the exploration and application of nanomaterial AI‐guided programmable assembly. Possessing flexible and malleable attributes, soft‐bodied robots exhibit distinct advantages when interacting with delicate or intricately shaped nanomaterials.^[^ ^112^ ^]^ Their capacity for agile movement and high‐precision manipulation allows for the delicate handling, accurate positioning, and efficient assembly of nanomaterials, making them particularly adept at constructing 3D nanostructures and conducting nanoscale surface modifications. Additionally, the gentle touch facilitated by soft robotics minimizes potential damage to nanomaterials, thus maintaining the integrity and quality of the assembly process.^[^ ^109^, ^113^ ^]^ By incorporating soft robotics, the efficiency and adaptability of nanomaterial assembly operations are markedly enhanced.

By combining shape‐variable liquid metal nanoparticles (LMNPs) with 3D printing technology, researchers have developed hybrid soft‐bodied robots that combine the best characteristics of soft and rigid components.^[^ ^114^ ^]^ This innovation not only enhances the robots' ability to perform tasks – for example, being able to achieve shape changes under near‐infrared light stimulation (i.e., 4D printing) – but also extends their range of applications, such as use as high‐precision grippers, bio‐inspired motors, or hand rehabilitation devices (Figure 15b). Specifically, the research demonstrates how a direct one‐step 3D printing approach, combined with a toolkit of functional materials with tuned mechanical properties and deformation capabilities, can be used to fabricate hybrid soft robotic systems capable of performing complex functions (e.g., rotational bistable devices, high‐precision gripping, bio‐inspired motors, and hand rehabilitation assistance). In summary, soft‐body robotics and its integration with advanced materials science provide new ways and tools to fabricate cutting‐edge nanodevices, while broadening the boundaries of nanoscience and technology applications and laying the foundation for breakthroughs in the field.

##### Laser Tweezer Technology in AI‐Guided Programmable Assembly

Laser tweezer technology represents a revolutionary approach to the manipulation of minute particles and nanoscale objects through the utilization of light pressure exerted by a focused laser beam.^[^ ^115^ ^]^ In recent years, the spatial resolution and manipulation accuracy of optical tweezers have been dramatically improved.^[^ ^116^ ^]^ By integrating customized optical tweezers with a state‐of‐the‐art device for dark‐field microscopy, nanoscale precision manipulation of AuNPs in different solvent environments has been achieved (Figure 15c).^[^ ^117^ ^]^ The study demonstrates that by integrating customized optical tweezers with state‐of‐the‐art dark‐field microscopy equipment, nanoscale precision manipulation of AuNPs in different solvent environments was achieved. Remarkably, they found that by tuning the laser to the nanoscale, precise control of the axial position of the particles could be achieved and the nanoparticles could be precisely localized in three dimensions.

This method is distinguished by its non‐contact nature, which allows for the precise movement and positioning of nanomaterials without the risk of physical damage, thereby ensuring high operational precision and reliability. With the aid of laser tweezers, scientists can construct intricate nanostructures with exceptional resolution and flexibility, facilitating the 3D assembly of nanomaterials (Figure 15d).^[^ ^116^ ^]^ Furthermore, laser tweezers offer the capability to modify surfaces and alter nanomaterials by finely tuning the laser's intensity and duration, which in turn can adjust the materials' physical and chemical properties. The adoption of laser tweezers technology has not only elevated the efficiency and quality of nanomaterial assembly but has also unlocked novel research and application pathways in nanotechnology, particularly in biomedicine, optoelectronic materials, and micro‐nano fabrication—sectors that exhibit vast potential for growth and innovation. The ongoing advancements in this technology are poised to significantly propel the development of nanoscience and technology, fostering the evolution and enhancement of associated industries.

##### 3D Printing Technology in AI‐Guided Programmable Assembly

The incorporation of 3D printing technology into the realm of nanomaterial AI‐guided programmable assembly heralds a new epoch of possibilities and transformations.^[^ ^118^ ^]^ Known formally as additive manufacturing, 3D printing technology excels in the precise formation of complex structures at microscopic and even nanoscopic levels. By sequentially depositing layers of nanomaterials, 3D printing enables the creation of nanostructures with elaborate geometries, offering greater flexibility and control over the design and fabrication process. Recent research^[^ ^119^ ^]^ showcases the Freeform Multi‐Material Assembly Process (FMAP), which blends 3D printing methods like fused filament fabrication (FFF) and direct ink writing (DIW) with freeform laser induction (FLI) to seamlessly assemble 3D engineered objects featuring complex geometries and multifunctionality. The FMAP platform not only facilitates the construction of structural assemblies but also streamlines the execution of AI‐guided programmable assembly within a single unit, reducing the necessity for multiple processing steps across different devices. In this process, FFF constructs structural assemblies, FLI transforms FFF‐printed materials into laser‐induced graphene (LIG) at predetermined locations, and DIW deposits precursor inks onto LIG electrodes for the subsequent laser‐induced formation of other functional materials like silver, iron, cobalt, nickel, and their oxides, yielding LIG‐based functional composites.

An additional advantage of 3D printing lies in its ability to integrate multifunctional materials, permitting the incorporation of diverse nanomaterials with distinct properties into a single composite structure, thereby generating materials with tailored functionalities. This technological breakthrough has not only revolutionized the precision and efficiency of nanomaterial assembly but has also opened up new frontiers for the application of nanotechnology across various sectors, including biomedicine, optoelectronics, and micro‐ and nano‐manufacturing. As a result, 3D printing technology is instrumental in advancing and diversifying the technological landscape, spurring innovation and development in interconnected fields.

##### AI and Big Data Analytics in AI‐Guided Programmable Assembly

The convergence of nanoscience and computational science, propelled by advancements in AI technologies, particularly machine learning algorithms, is poised to catalyze groundbreaking innovations in AI‐guided programmable assembly.^[^ ^86^, ^120^ ^]^ With enhancements in big data processing capabilities and algorithm optimization, machine learning models are emerging as formidable tools for delving into AI‐guided assemblies,^[^ ^121^ ^]^ offering the potential to forecast the behavior of nanocrystalline superstructures and their physicochemical properties across varying conditions with unparalleled speed and precision.^[^ ^122^ ^]^ This not only holds the promise of significantly diminishing the material resources and time investments entailed by traditional experimental methods but also facilitates a deeper comprehension of the unique attributes of novel materials.^[^ ^123^ ^]^ Notably, the application of big data analytics and machine learning algorithms can expedite the screening of optimal AI‐guided programmable assembly combinations, thereby curtailing the research and development cycle.

More importantly, research leveraging advanced methodologies such as deep learning and reinforcement learning holds the potential to achieve precise regulation of the dynamic behavior of AI‐guided programmable assembly smart superstructures. Through the application of these cutting‐edge technologies, researchers are actively engaged in developing intelligent nanosystems capable of autonomously adapting to external environmental changes.^[^ ^124^ ^]^ By training neural networks, these systems are endowed with the capability to automatically adjust interactions among nanoparticles across diverse conditions, thereby enabling the entire structural framework to demonstrate self‐adaptability. Such intelligent control mechanisms not only exhibit swift responses to external stimuli but also undertake intricate tasks, attaining a genuinely intelligent status. As AI and big data analytics continue to evolve, their integration with AI‐guided programmable assembly techniques will not only accelerate research in nanomaterials but also help bridge the gap between theoretical predictions and real‐world applications. The ability to simulate, predict, and optimize nanostructure behavior before physical fabrication will drastically reduce costs and improve the efficiency of the development process. Furthermore, the deployment of AI‐driven automated systems for assembly could lead to entirely new manufacturing paradigms, where nanomaterials are created and adapted in response to changing conditions in real time, thus enabling more sustainable and customizable solutions across a wide array of industries. Subsequent investigations will delve deeper into harnessing sophisticated computational tools and technological approaches to confer these superstructures with a broader spectrum of functionalities, positioning them to play pivotal roles across various domains such as biomedicine, information storage, energy conversion, and beyond.

#### 4.2.2 Application Prospect of AI‐Guided Programmable Assembly

As advancements in AI‐guided programmable assembly persist and evolve, there is a compelling rationale to anticipate that smart nanoparticle superstructures will carve out a distinctive niche in various domains in the foreseeable future. The precise control over the assembly of nanoparticles allows for the creation of materials that can be tailored for specific functionalities. These innovations are poised to revolutionize fields such as information storage, sensing technology, medical and health sciences, quantum information, and other cutting‐edge realms of science and technology.

##### Information Storage

At the forefront of information storage technology, smart nanoparticle superstructures are leading a revolution. By precisely controlling the position and arrangement of nanoparticles, this technology not only achieves a significant increase in data storage density but also opens up innovative paths comparable to DNA storage technology. Thanks to the unique properties of nanoparticle sequences, researchers are able to dramatically expand data capacity and ensure stable long‐term preservation of data. Advances in AI‐guided self‐assembly technology have further pushed the boundaries of this field, paving the way for the development of novel storage mechanisms such as phase change memory (PCM) and resistive random access memory (ReRAM). These innovative storage solutions are not only expected to surpass traditional magnetic and optical storage media, achieving breakthroughs in storage capacity, data read/write rates, and device lifetimes, but also, due to the tiny dimensions of their underlying components – nanoparticles – can theoretically enable massive amounts of data storage in a very small space, thus effectively responding to the growing global demand for data storage. In addition, nanoparticle‐based information storage technologies demonstrate other advantages: lower energy consumption and higher physical durability, for example. This gives them great potential for use in data centers, personal computing devices, and even the emerging Internet of Things (IoT) applications.

However, transitioning this technology from the laboratory phase to real‐world applications encounters several hurdles, including the intricacy of the manufacturing process, cost‐effectiveness evaluations, and the assurance of long‐term stability and reliability. Despite these challenges, ongoing research and technological progress are anticipated to propel smart nanoparticle superstructures into a pivotal position in the realm of information storage, potentially heralding an era of ultra‐high‐density data storage in the future.

##### Sensing Technology

The binary AI‐guided programmable assembly technology of nanoparticles holds immense promise in sensing and detection applications. By precisely controlling the assembly of nanoparticles, it is possible to address issues prevalent in traditional sensors and unlock new possibilities for enhanced sensitivity and customized sensing applications. One of the key advantages lies in the ability to create composites with expedited electron transport paths, leading to significantly improved response times to target analytes. This addresses the challenge of slow response times in traditional sensors. Furthermore, by designing specific nanoparticle assemblies, it becomes feasible to enhance the sensor's selectivity to recognize particular molecules or ions, thereby reducing background interference in complex environments. Moreover, nanoparticles can form stable structures that ensure high performance even under harsh conditions, thereby enhancing long‐term sensor stability. In applications such as detecting trace analytes like volatile organic compounds (VOCs) in exhaled breath, nanoparticles' interactions, such as electron transfer and energy migration, can drastically boost detection sensitivity, enabling the capture of even minute concentrations effectively.

The technology's flexibility allows for optimal design for various scenarios, especially in healthcare applications like exhaled gas analysis. The binary AI‐guided programmable assembly of nanoparticles offers an efficient platform for enhancing responses to biomarkers, enabling early disease detection without complex sample pre‐processing. Furthermore, this technology boasts a range of advanced features such as timed detection, region‐specific detection, simultaneous detection, and step‐by‐step detection. By programming the behavior of nanoparticles, researchers can craft sensors that trigger responses at specific time points, facilitating timed detection. This capability is crucial for monitoring cyclical changes like breathing patterns or drug metabolism, ensuring the acquisition of precise data at the most opportune moments. Leveraging the localization and alignment attributes of nanoparticles, it is feasible to develop sensors that are selectively sensitive within designated areas, enabling fixed‐area detection. This precision is particularly valuable for applications necessitating accurate localization, such as environmental monitoring or food quality control, to diminish background interference and enhance targeting and precision. Through the synergistic interactions between nanoparticles, the creation of sensors capable of simultaneously detecting multiple parameters or analytes becomes achievable, enhancing detection efficiency and data integrity. Additionally, by adjusting the nanoparticle ratio and assembly method, sensors can be tailored to gradually respond to different analytes in a predetermined sequence to achieve stepwise detection. This method is especially suited for the systematic resolution of complex samples, ensuring the accurate identification and quantification of each component.

##### Medical and Health Sciences

AI‐guided programmable assembly technologies play a crucial role in healthcare, addressing limitations in traditional approaches and offering the potential for high‐precision, versatile, and tailored medical solutions. Conventional drug delivery systems often encounter challenges like imprecise targeting, significant side effects, and unregulated drug release. Similarly, tissue‐engineered materials may struggle due to issues related to biocompatibility and structural functionality. AI‐guided programmable assembly technology has the capacity to effectively overcome these shortcomings by creating smart drug delivery systems through meticulous customization of nanocarrier surfaces, particularly leveraging DNA sequences. In the realm of smart drug delivery systems, meticulously crafted nanoparticles can release medications exclusively in designated cells or tissues, significantly enhancing efficacy and minimizing adverse effects. Moreover, smart drug release systems can react to external stimuli such as temperature, pH, or specific molecules, enabling precise and controlled drug release, thereby facilitating more accurate and efficient therapies. Regarding tissue engineering, AI‐guided programmable assembly technology aids in producing engineered materials that emulate the characteristics of natural tissues. These technological advancements are crucial for developing artificial organs, skin substitutes, and other biomedical implants, advancing medical science, and offering safer and more effective treatment options for patients.

AI‐guided programmable assembly technology not only overcomes critical obstacles in traditional medical treatments, such as non‐targeted drug delivery and biocompatibility challenges in tissue‐engineered materials, but also introduces features like targeted drug delivery, smart release mechanisms, and advanced tissue engineering. These advancements significantly enhance the effectiveness and safety of medical interventions, paving the way for personalized medicine, disease treatment, and regenerative medicine. With ongoing research and technological progress, there is a strong belief that AI‐guided programmable assembly technology will continue to lead healthcare innovation and development, making substantial contributions to human health.

##### Quantum Information

Nanoparticle binary AI‐guided programmable assembly technology is a revolutionary advancement in the realm of quantum communications, holding significant promise for the development of quantum light sources. Traditional quantum light sources and optoelectronic devices encounter several challenges, including low photon emission efficiency, high noise levels, directional emission difficulties, and the inability to produce single‐photon pairs or entangled photon pairs on demand. These limitations constrain the effectiveness and reliability of quantum communication technologies like Quantum Key Distribution (QKD) and hinder the growth and advancement of quantum networks. For instance, research by Li et al.^[^ ^20c^ ^]^ illustrated that an atomically ordered assembly of CsPbBr_3_ chalcogenide nanocubes forms a superlattice structure showcasing a multiscale coherent state. These structured assemblies can be scaled up to meter‐sized panels as micropixel luminescent layers for primary color photon emitters. The resultant layered assemblies not only enhance optoelectronic device performance but also enable the creation of highly efficient, directional quantum light sources. Such quantum light sources facilitate the on‐demand generation of single‐photon or entangled‐photon pairs, pivotal for realizing quantum communication technologies like quantum key distribution. Moreover, these advanced quantum light sources propel the advancement of quantum networks, forming the backbone of infrastructure linking quantum devices such as quantum computers, memories, and sensors. Leveraging high‐efficiency, low‐noise quantum light sources ensures stable quantum information transmission, bolstering the establishment of extensive quantum networks.

Compared to conventional methods, nanoparticle binary AI‐guided programmable assembly technology can overcome several key obstacles to improve photon emission efficiency, reduce noise levels, enable directional emission, and generate specific types of photon pairs on demand. Conventional quantum light sources are limited by low photon emission rates and unavoidable background noise, which affects the quality of quantum information; whereas, through well‐designed nanoparticle assembly, not only can the photon emission efficiency be dramatically increased, but also unwanted spontaneous radiation and other forms of noise can be reduced to improve signal purity. Meanwhile, the new assembly technique makes the quantum light source highly directional, ensuring that the photons are efficiently directed to the target receiver, and can be customized to generate single‐photon or entangled‐photon pairs by adjusting the ratio and arrangement of the nanoparticles to satisfy different quantum communication needs.

The nanoparticle binary AI‐guided programmable assembly technology not only solves the key shortcomings of conventional quantum light sources and optoelectronic devices but also introduces new features such as high efficiency, low noise, directional emission, and on‐demand photon pair generation. These advances have greatly enhanced the effectiveness and safety of quantum communication, paving the way for future personalized medicine, disease treatment, and regenerative medicine. With in‐depth research and technological advances, this technology will continue to lead innovation and development in the field of quantum communications and make greater contributions to the advancement of human science and technology. In the future, with the application of these advanced light sources, quantum networks will become even more mature and powerful, revolutionizing fields such as quantum computing and secure communications.

## 5 Conclusion

This review provides a comprehensive overview of the evolution of colloidal nanoparticle self‐assembly and its binary co‐assembly technology from its infancy to maturity, emphasizing that the field has moved beyond the early stage of simple inorganic nanoparticle assembly into a new era of being able to build complex structures and exhibit novel properties. Currently, the self‐assembly of colloidal nanoparticles and their binary co‐assembly technology are in a critical period of rapid development, and their future applications are promising. With the deepening understanding of the interaction mechanisms between nanoparticles, this field not only provides a solid theoretical basis for designing nanostructures with specific functions but also enables a high degree of control over the arrangement of nanoparticles by precisely adjusting their parameters such as surface chemistry, size, and morphology, thus creating superstructures with excellent physicochemical properties. The above advances pave the way for introducing the concept of AI‐guided programmable assembly into this research field, laying an important research foundation for exploring the design and synthesis of novel materials. In particular, this paper highlights the feasibility and potential technological means of AI‐guided programmable assembly and provides an outlook on its application. AI‐guided programmable assembly technology allows scientists to manipulate the structure formation process at the nanoscale with greater precision and flexibility, leading to the development of new materials with customized functionalities. This not only enhances the freedom of material design but also paves the way for innovative applications in a variety of fields, including smart materials, information storage, biomedicine, and energy. In addition, with the deepening of multidisciplinary cross‐collaboration, we can expect that this field will continue to produce more innovations that will further expand its application scope and enhance its practical value, providing strong support for solving the many challenges faced by today's society.

Looking ahead, the field of smart AI‐guided nanomaterials is filled with both opportunities and challenges. To advance this field, it is essential to delve into various aspects. At the basic research level, a deeper understanding of the interaction mechanisms of nanoparticles is paramount. This involves not only comprehending the physical and chemical properties of nanoparticles but also their behavioral patterns in different environments. Accurate knowledge in this area can aid in developing precise self‐assembly models and prediction algorithms, thereby enhancing the success rate and controllability of the self‐assembly process. Another critical aspect is the realization of large‐scale production and widespread application of smart nanomaterials. While laboratory‐level self‐assembly technology is relatively mature, challenges persist in extending these materials to industrial production. Ensuring consistency, stability, and cost reduction are key hurdles that need to be addressed. Future research should focus on developing more efficient, reliable, and economically viable self‐assembly methods. With the progression of nanotechnology, the development of real‐time monitoring and feedback regulation systems has become increasingly important. This entails dynamically regulating the assembly process of nanoparticles during experiments. By integrating advanced characterization techniques with real‐time data analysis, the self‐assembly process can be accurately controlled to optimize the assembly effect and rectify any deviations promptly to ensure the quality of the final product. Moreover, interdisciplinary collaboration and technological innovation are indispensable for the advancement of smart AI‐guided superstructured colloidal nanoparticles. Enhanced communication and cooperation across natural sciences such as physics, chemistry, biology, and integration with fields like engineering and computer science are necessary to explore new ways of designing, synthesizing, and applying new materials. By amalgamating multidisciplinary knowledge and technology, innovative solutions can be expected, expanding the application scope of smart nanomaterials. In conclusion, continuous technological innovation and interdisciplinary collaboration are vital for overcoming challenges and achieving breakthroughs in the development of smart nanomaterials amidst their complexity and diversity.

The introduction of AI‐guided programmable assembly ideas into the field of nanocrystalline self‐assembly and the realization of a shift in self‐assembly from traditional passive assembly to active intelligent design not only reflects a major theoretical breakthrough in nanomaterials science but also demonstrates its great potential in practical applications. This review aims to stimulate the attention and commitment of more researchers through an in‐depth discussion of the progress in the field of colloidal nanoparticle self‐assembly and to jointly promote the progress and development of nanoscience and technology, to bring greater benefits to human society.

## Conflict of Interest

The authors declare no conflict of interest.
